# Supplementary material for: c-Myc inactivation of p53 through the pan-cancer lncRNA MILIP drives cancer pathogenesis
Source: Nat Commun. 2020 Oct 5;11:4980. doi: 10.1038/s41467-020-18735-8 (PMC7536215; doi:10.1038/s41467-020-18735-8)
Supplement: Supplementary file 1 — Supplementary Information [file 41467_2020_18735_MOESM1_ESM.pdf]

c-Myc inactivation of p53 through the pan-cancer lncRNA MILIP drives cancer pathogenesis

Feng et al.

Supplementary Figure 1

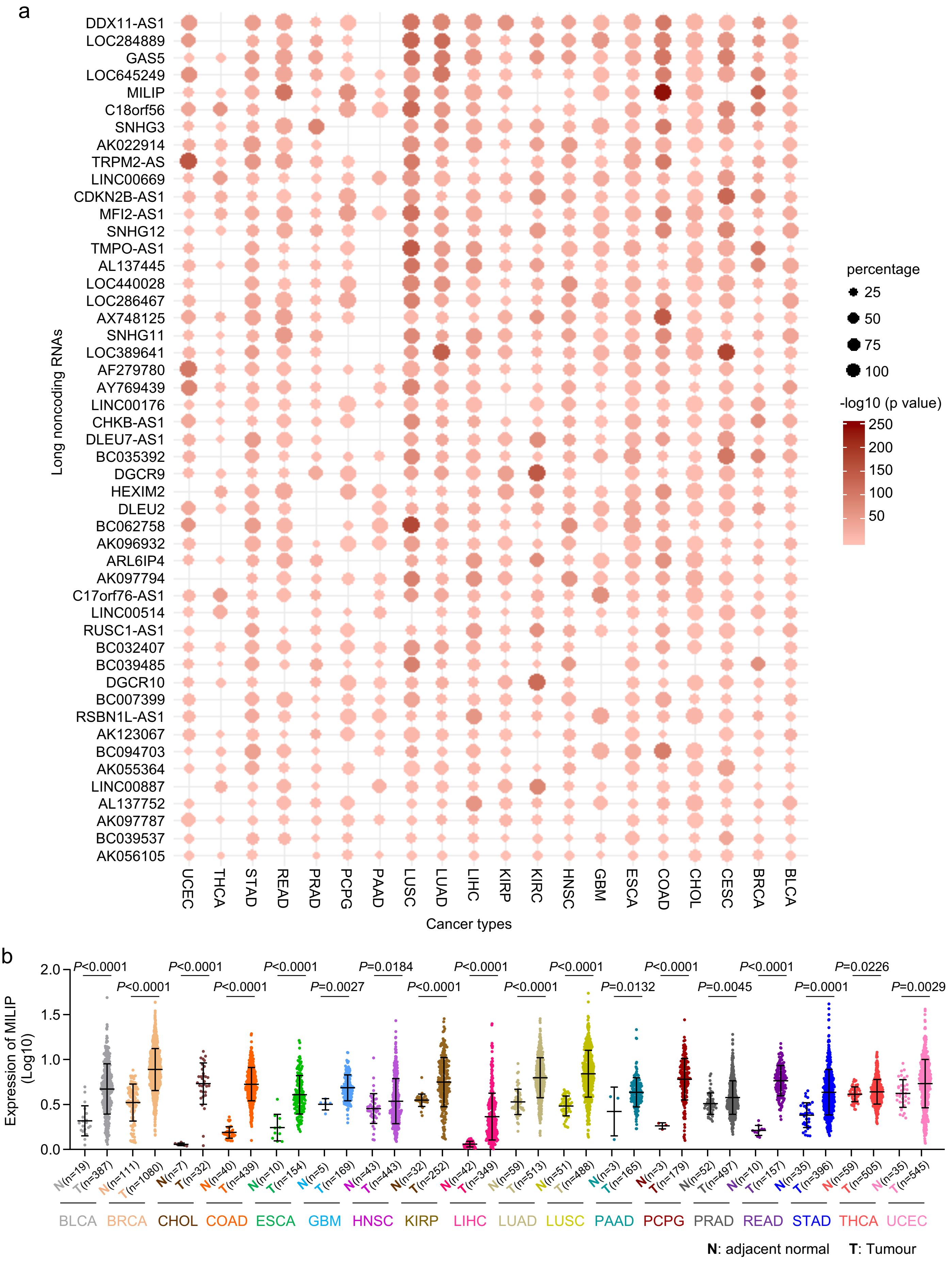

Supplementary Figure 1. MILIP is a pan-cancer-associated lncRNA

a, Identification of pan-cancer-associated lncRNAs through analysis of the lncRNA expression data in the TCGA. LncRNAs that are increased in at least 18 out of 20 cancer types are depicted. Each dot represents the expression levels of a lncRNA in a type of cancer tissues relative to corresponding normal tissues. (BLCA: bladder urothelial carcinoma; BRCA: breast invasive carcinoma; CESC: cervical squamous cell carcinoma and endocervical adenocarcinoma; CHOL: cholangiocarcinoma; COAD: colon adenocarcinoma; ESCA: esophageal carcinoma; GBM: glioblastoma multiforme; HNSC: head and neck squamous cell carcinoma; KIRC: kidney renal clear cell carcinoma; KIRP: kidney renal papillary cell carcinoma; LIHC: liver hepatocellular carcinoma; LUAD: lung adenocarcinoma; LUSC: lung squamous cell carcinoma; PAAD: pancreatic adenocarcinoma; PCPG: pheochromocytoma and paraganglioma; PRAD: prostate adenocarcinoma; READ: rectum adenocarcinoma; STAD: stomach adenocarcinoma; THCA: thyroid carcinoma; UCEC: uterine corpus endometrial carcinoma). **b,** MILIP upregulation in diverse cancer types compared with corresponding normal tissues as revealed by analysis of the lncRNA expression data in the TCGA. Data are mean ± s.d.; two-tailed Student's *t*-test. Source data of Supplementary Fig. 1b are provided as a Source Data file.

Supplementary Figure 2

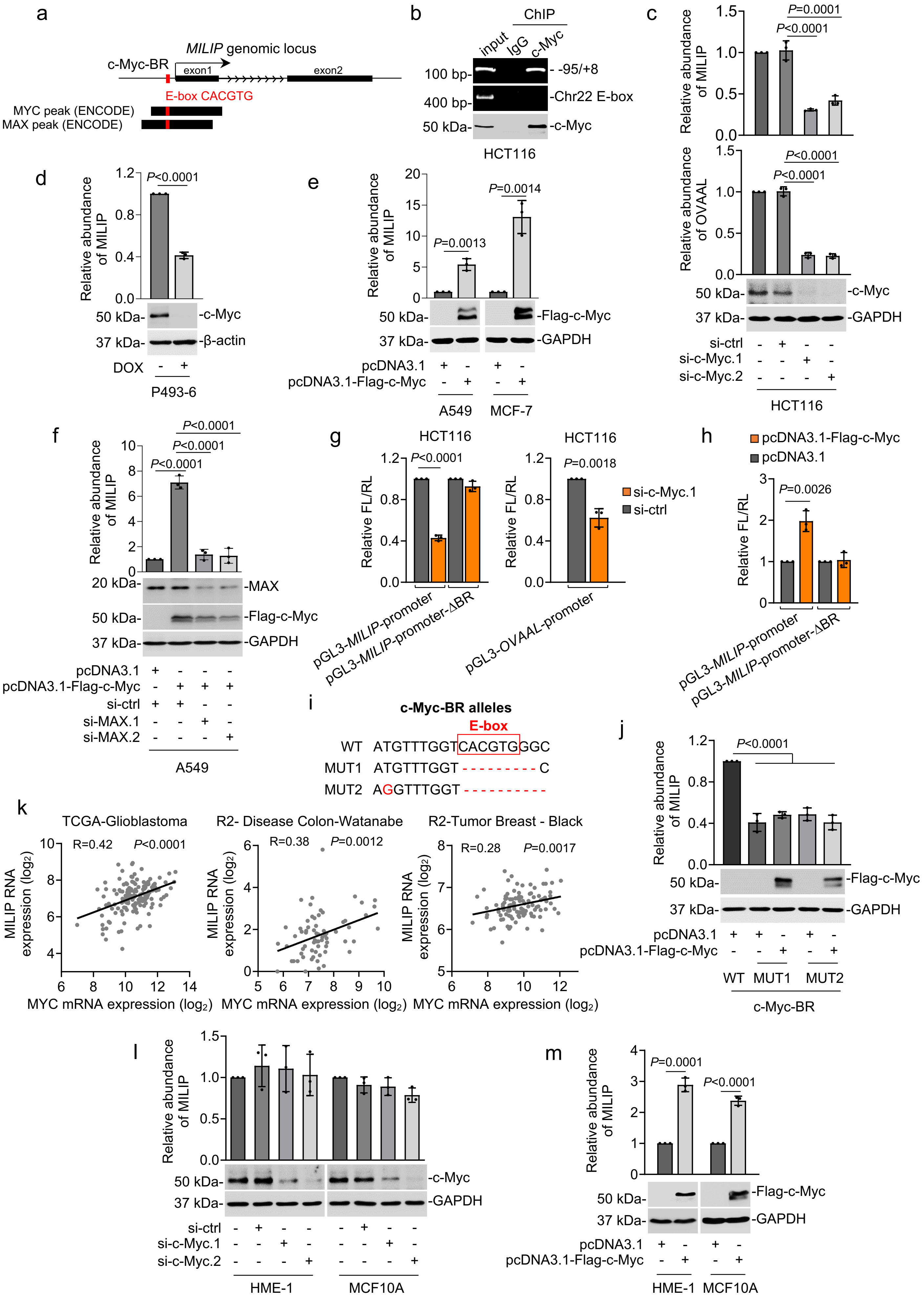

Supplementary Figure 2. c-Myc regulates MILIP expression

a, A schematic illustration of a consensus c-Myc binding region (c-Myc-BR) and ChIP-seq peaks of Myc and Max protein in *MILIP* promoter from ENCODE. ChIP-seq: Chromatin Immunoprecipitation Sequencing; ENCODE: Encyclopedia of DNA Elements. **b,** c-Myc bound to *MILIP* promoter in HCT116 cells. An E-box motif, not associated with MYC target genes on Chr22, was used as a negative control. Data shown represent three independent experiments. ChIP, chromatin immunoprecipitation. **c,** c-Myc silencing downregulated MILIP expression in HCT116 cells. c-Myc responsive lncRNA OVAAL was used as a positive control. Data are mean ± s.d.; n = 3 independent experiments, One-way ANOVA followed by Tukey's multiple comparisons test. **d,** Induced silencing of c-Myc downregulated MILIP expression. Data are mean ± s.d.; n = 3 independent experiments, two-tailed Student's *t*-test. **e, f,** Overexpression of c-Myc upregulated MILIP expression (e), which was abolished by knockdown of Max (f). Data are representatives or mean ± s.d.; n = 3 independent experiments, two-tailed Student's *t*-test. **g,** c-Myc silencing reduced the activity of reporters with intact c-Myc binding region (BR) of *MILIP* promoter but not that with the c-Myc-BR deleted (ΔBR) in HCT116 cells. c-Myc responsive lncRNA OVAAL was used as a positive control. Data are mean ± s.d.; n = 3 independent experiments, two-tailed Student's *t*-test. **h,** Overexpression of c-Myc increased the activity of reporters with intact c-Myc BR of *MILIP* promoter but not that with c-Myc-BR deleted (ΔBR) in A549 cells. Data are mean ± s.d.; n = 3 independent experiments, two-tailed Student's *t*-test. **i,** c-Myc-BR wild-type (WT) and mutant (MUT) alleles in *MILIP* promoter region generated by CRISPR/Cas9 were examined by Sanger sequencing. **j,** CRISPR/Cas9-mediated deletion mutagenesis at the c-Myc-BR in endogenous *MILIP* gene promoter reduced MILIP expression, which was not rescued by c-Myc overexpression. Data are representatives or mean ± s.d.; n = 3 independent experiments, One-way ANOVA followed by Tukey's multiple comparisons test. **k,** Linear regression analysis of the relationship between MILIP and *MYC* RNA expression in cancer datasets derived from the R2 Genomics Analysis and Visualization Platform (R2). Two-tailed Pearson correlation coefficient test. **l,** c-Myc silencing did not alter MILIP expression in normal human epithelial cell lines MCF10A and HME-1. Data are mean ± s.d.; n = 3 independent experiments. **m,** Overexpression of c-Myc increased MILIP expression in normal human epithelial cell lines MCF10A and HME-1. Data are mean ± s.d.; n = 3 independent experiments, two-tailed Student's *t*-test. Source data of Supplementary Figs. 2b-h, j-m are provided as a Source Data file.

Supplementary Figure 3

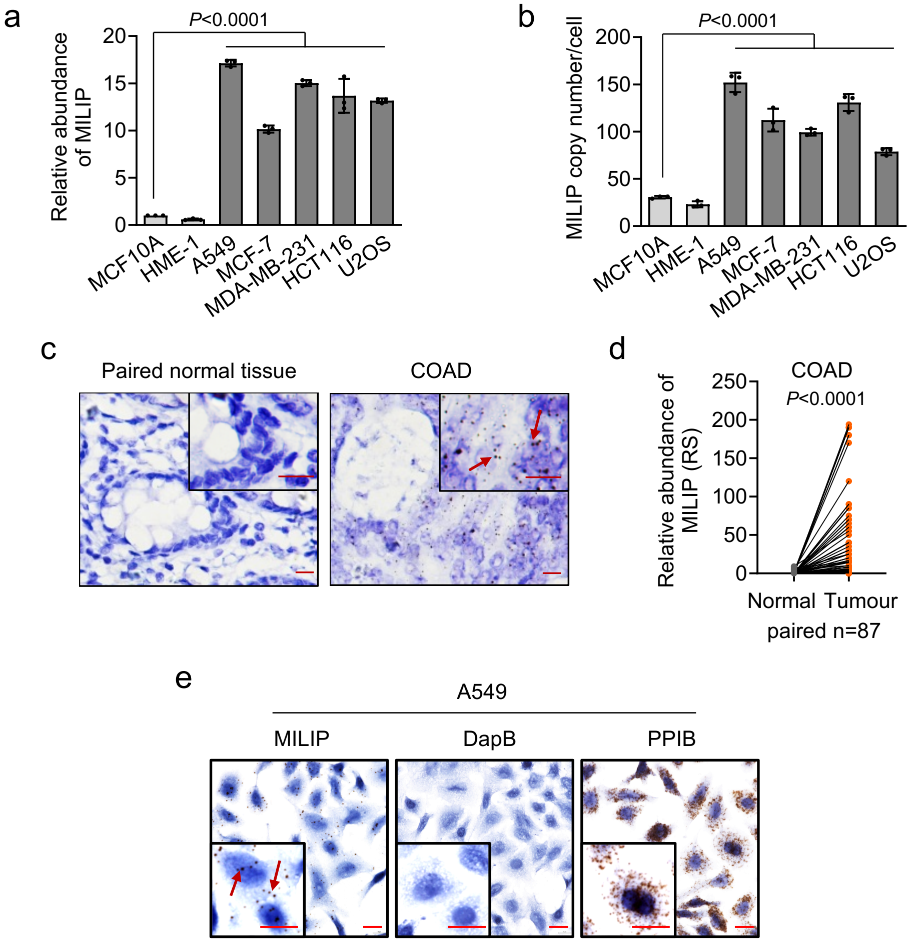

Supplementary Figure 3. MILIP is highly expressed in diverse cancer types

a, b, Higher expression levels (a) and more copy numbers (b) of MILIP in A549 (lung adenocarcinoma), MCF-7 (breast adenocarcinoma), MDA-MB-231 (breast adenocarcinoma), HCT116 (colorectal adenocarcinoma), and U2OS (Osteosarcoma) cell lines in comparison with normal human epithelial cell lines MCF10A and HME-1 were determined by qPCR. Data are mean ± s.d.; n = 3 independent experiments, One-way ANOVA followed by Tukey's multiple comparisons test. **c,** Representative microscopic photographs of *in situ* hybridization (ISH) analysis of MILIP expression in formalin-fixed paraffin-embedded (FFPE) colon adenocarcinoma (COAD; n = 87 biologically independent samples) compared with paired adjacent normal tissues. Scale bar, 5 µm. **d,** Quantitation of MILIP expression as detected in FFPE COAD in comparison with paired adjacent normal tissues. RS: reactive score. Two-tailed Student's *t*-test. **e,** *In situ* hybridization (ISH) analysis of MILIP expression in A549 cells grown on coverslips. ISH analysis of DapB and PPIB RNAs was included as a negative and a positive control, respectively. Scale bar, 25 µm. Data shown represent three independent experiments. Source data of Supplementary Figs. 3a, b, d are provided as a Source Data file.

Supplementary Figure 4

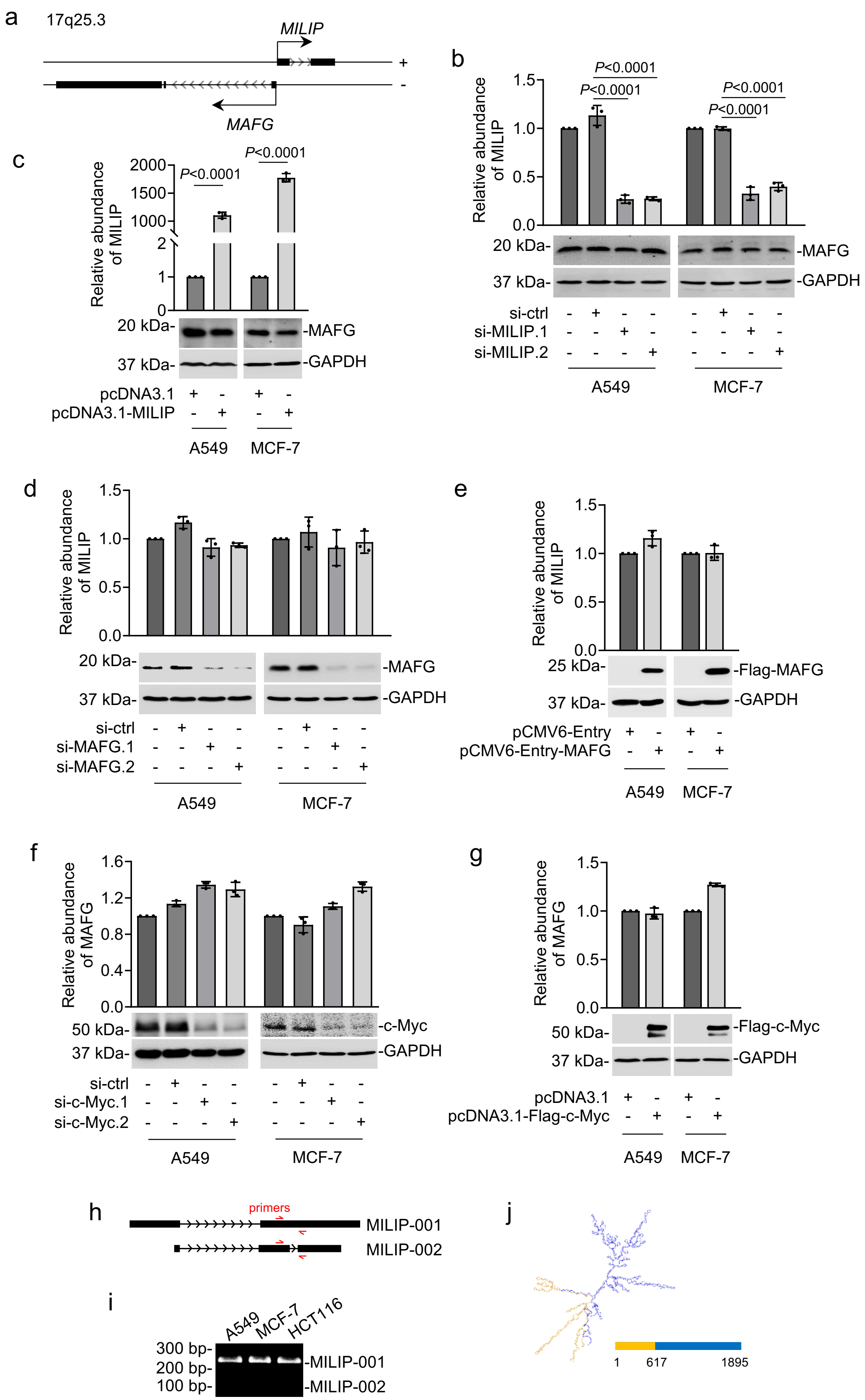

Supplementary Figure 4. MILIP does not influence the expression of MAFG

a, A schematic illustration of the genomic location of *MILIP* and *MAFG*. **b, c,** Neither knockdown (b) nor overexpression (c) of MILIP influences MAFG expression in A549 and MCF-7 cells. Data are representatives or mean ± s.d.; n = 3 independent experiments, One-way ANOVA followed by Tukey's multiple comparisons test (b), two-tailed Student's *t*-test (c). **d, e,** Neither knockdown (d) nor overexpression (e) of MAFG influences MILIP expression in A549 and MCF-7 cells. Data are representatives or mean ± s.d.; n = 3 independent experiments. **f, g,** Neither knockdown (f) nor overexpression (g) of c-Myc influences MAFG expression in A549 and MCF-7 cells. Data are representatives or mean ± s.d.; n = 3 independent experiments. **h,** A schematic illustration of two identified isoforms of MILIP from Vega Genome Browser. The indicated primers were used to identify MILIP-001 and MILIP-002. **i,** MILIP-001 but not MILIP-002 is the major isoform of *MILIP* gene in A547, MCF-7 and HCT116 cells as detected using PCR. Data shown represent three independent experiments. **j,** MILIP secondary structure is predicted based on minimum free energy algorithm. Source data of Supplementary Figs. 4b-g, i are provided as a Source Data file.

Supplementary Figure 5

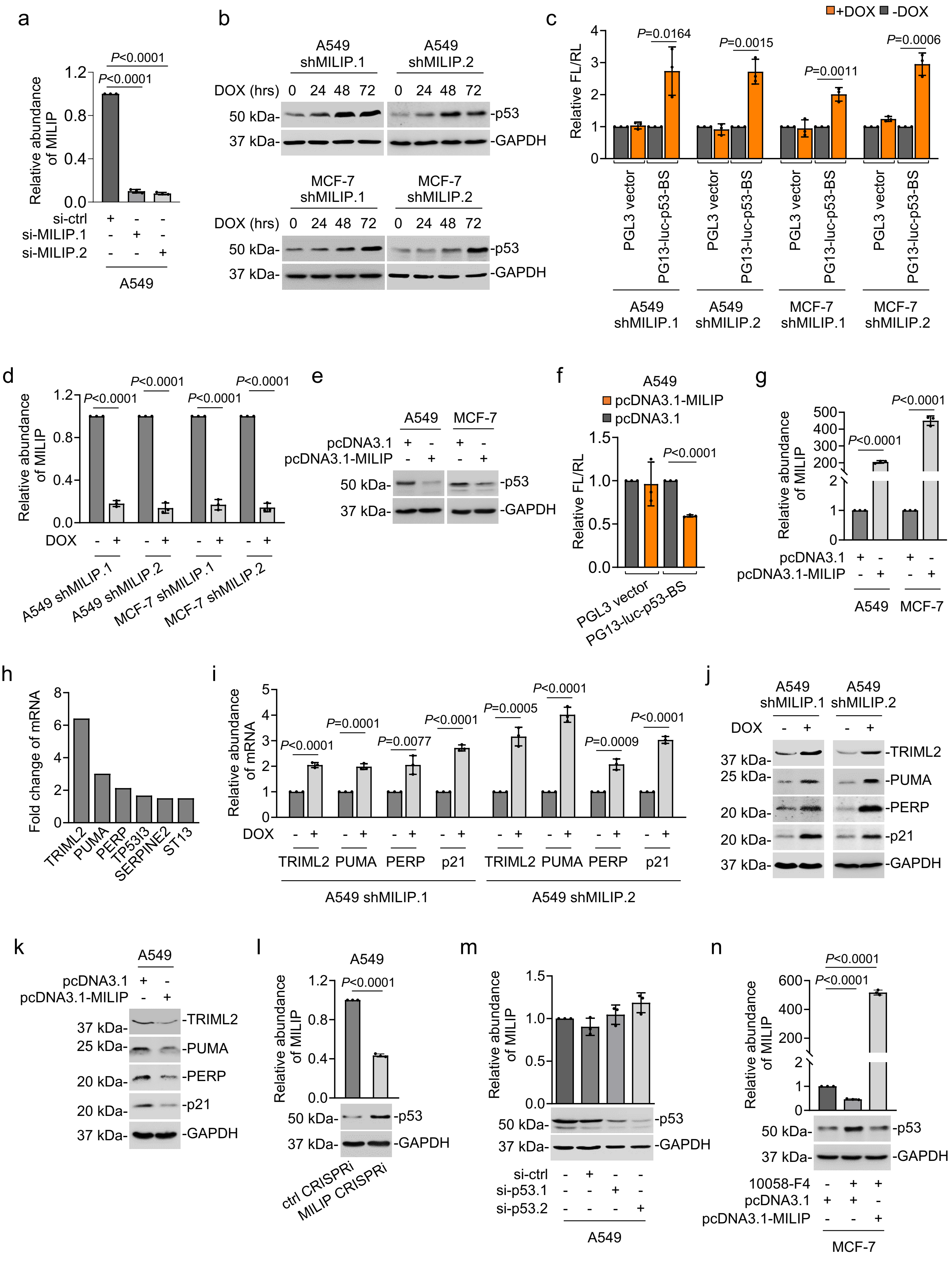

Supplementary Figure 5. MILIP represses p53

a, MILIP levels in A549 cells transfected with the control siRNAs (si-ctrl), MILIP siRNA 1 (si-MILIP.1) or MILIP siRNA 2 (si-MILIP.2) were measured by qPCR. Data are representatives or mean ± s.d.; n = 3 independent experiments, One-way ANOVA followed by Tukey's multiple comparisons test. **b-d,** p53 expression levels were increased in a time-dependent manner (b) and the p53 luciferase reporter (PG13-luc-p53-binding sites (BS)) activities were enhanced (c) by induced knockdown of MILIP (d) in cells carrying inducible MILIP shRNA.1 or MILIP shRNA.2 in response to treatment with doxycycline (DOX, 200 ng/ml). Data are representatives or mean ± s.d.; n = 3 independent experiments, two-tailed Student's *t*-test. FL: Firefly luciferase activity; RL: Renilla luciferase activity. **e-g,** p53 expression levels were decreased (e) and the p53 luciferase reporter (PG13-luc-p53-BS) activities were reduced (f) by overexpression of MILIP (g). Data are representatives or mean ± s.d.; n = 3 independent experiments, two-tailed Student's *t*-test. **h,** Knockdown of MILIP upregulated the p53 downstream targets tripartite Motif Family Like 2 (TRIML2), p53 upregulated modulator of apoptosis (PUMA), p53 apoptosis effector related to PMP-22 (PERP), Tumour Protein P53 Inducible Protein 3 (TP53I3), Serpin Family E Member 2 (SERPINE2) and suppression of tumorigenicity 13 (ST13) at the mRNA level detected using RNA-seq. n = 1 experiment. **i,** Induced knockdown of MILIP upregulated the p53 downstream targets TRIML2, PUMA, PERP and p21 at the mRNA level detected using qPCR. Data are mean ± s.d.; n = 3 independent experiments, two-tailed Student's *t*-test. **j, k,** Induced knockdown of MILIP upregulated (j) whereas overexpression of MILIP downregulated (k) the p53 downstream targets TRIML2, PUMA, PERP and p21 at the protein level. Data shown represent three independent experiments. **l,** Inhibition of MILIP transcription by sgRNA-mediated CRISPR interference increased the expression of p53. Data are representatives or mean ± s.d.; n = 3 independent experiments, two-tailed Student's *t*-test. **m,** Knockdown of p53 did not alter the expression of MILIP. Data are representatives or mean ± s.d.; n = 3 independent experiments. **n,** Treatment with the c-Myc inhibitor 10058-F4 upregulated p53 expression, which was diminished by MILIP overexpression. Data are representatives or mean ± s.d.; n = 3 independent experiments, One-way ANOVA followed by Tukey's multiple comparisons test. Source data of Supplementary Figs. 5a-n are provided as a Source Data file.

Supplementary Figure 6

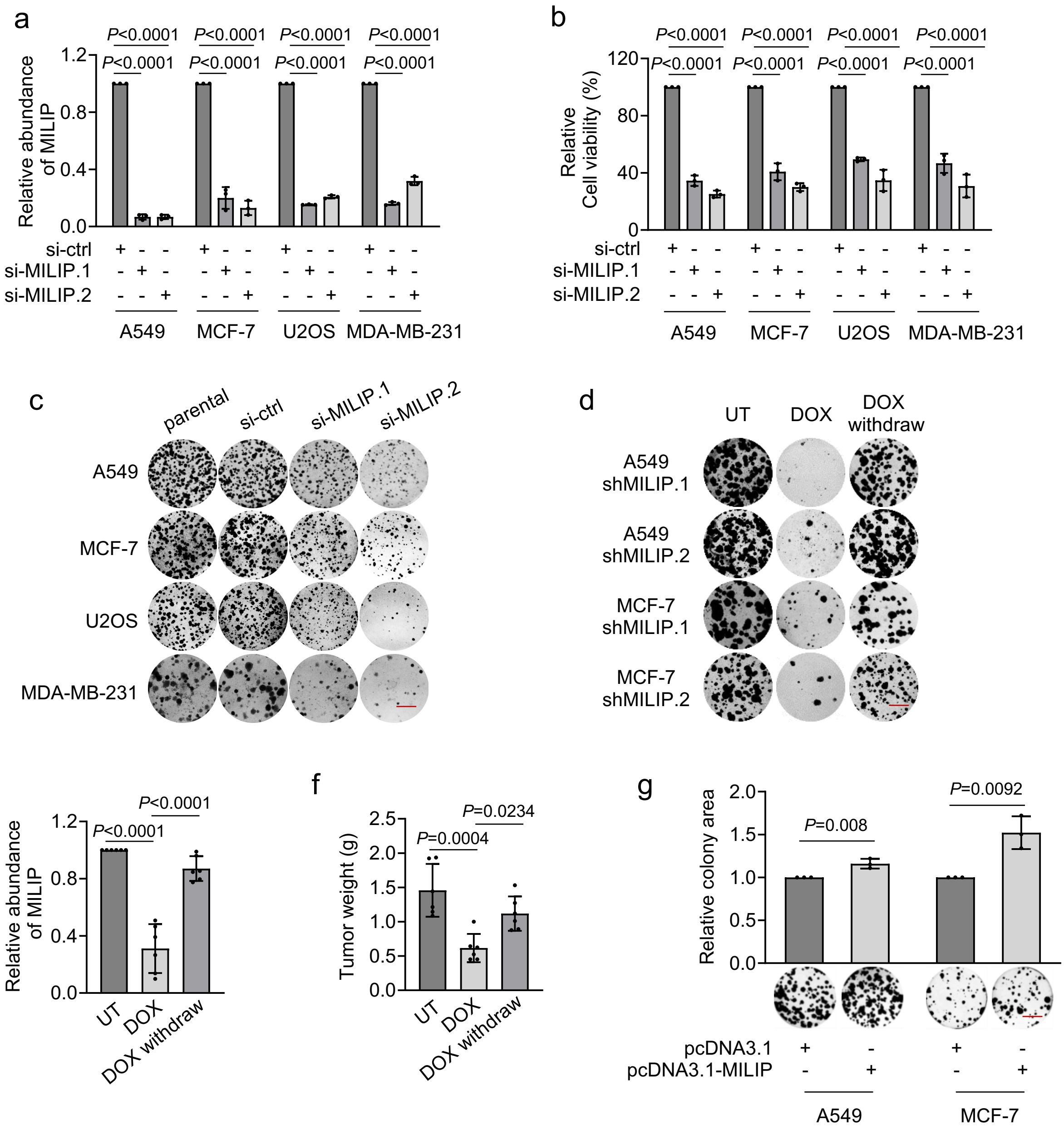

Supplementary Figure 6. MILIP promotes cancer cell viability and tumourigenicity

a-c, Silencing of MILIP (a) reduced the viability (b) and the clonogenicity (c) of A549, MCF-7, U2OS, and MDA-MB-231 cells. Data are representatives or mean ± s.d.; n = 3 independent experiments, One-way ANOVA followed by Tukey's multiple comparisons test. **d,** Induced knockdown of MILIP reduced clonogenicity of A549 and MCF-7 cells, which was reversed by DOX withdrawal. Scale bar, 1 cm. Data shown represent three independent experiments. DOX: 200 ng/ml. **e, f,** Induced knockdown of MILIP (e) reduced A549 xenografted tumour weights, which were reversed by DOX withdrawal in nu/nu mice (f). Data are mean ± s.d.; n = 6 mice per group, One-way ANOVA followed by Tukey's multiple comparisons test. DOX: 2 mg/ml supplemented with 10 mg/ml sucrose in drinking water. **g,** Overexpression of MILIP moderately increased the clonogenicity of A549 and MCF-7 cells. Data are representatives or mean ± s.d.; n = 3 independent experiments, two-tailed Student's *t*-test. Source data of Supplementary Figs. 6a, b, e-g are provided as a Source Data file.

Supplementary Figure 7

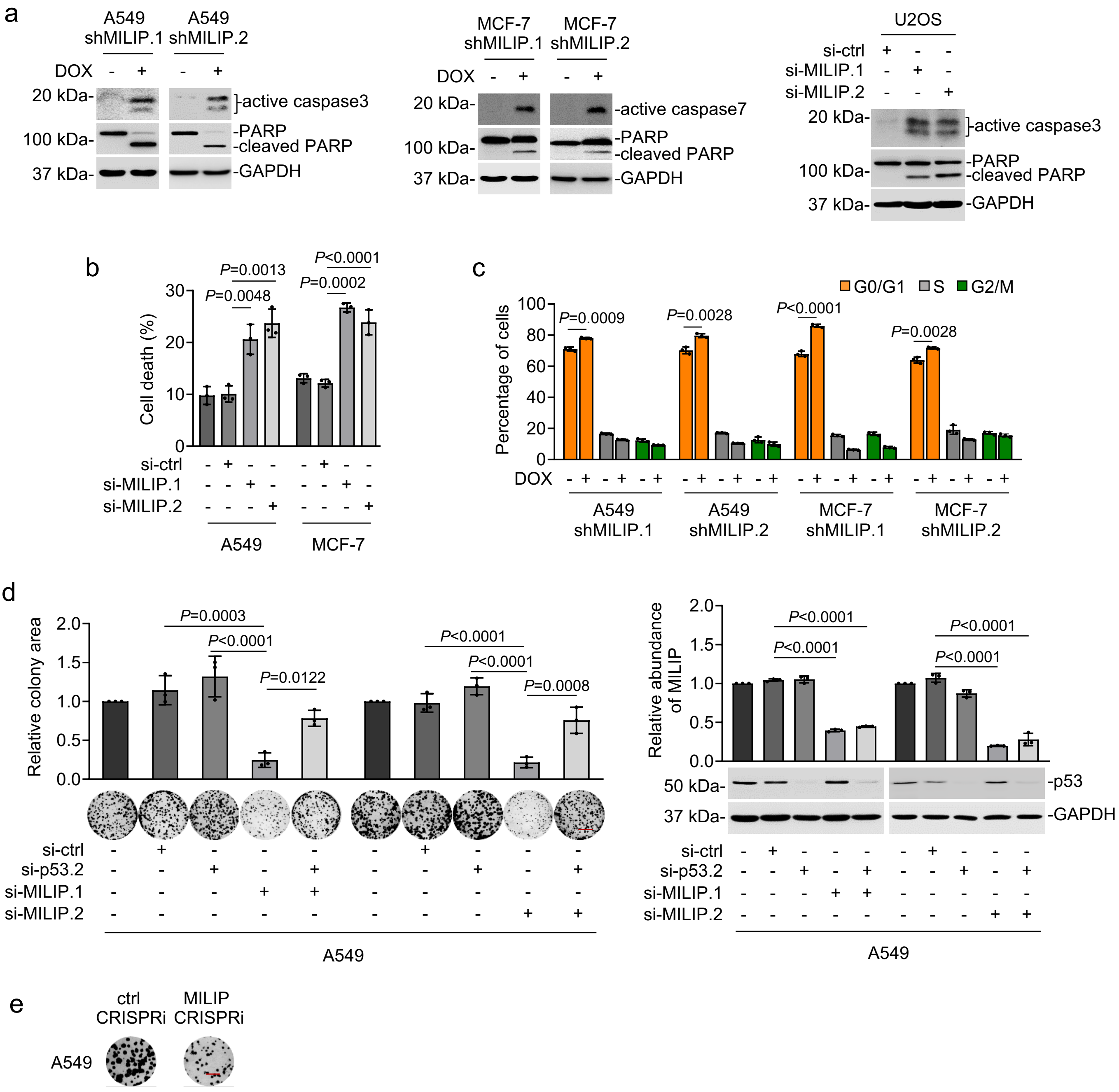

Supplementary Figure 7. MILIP promotes cancer cell viability via suppressing p53

a, b, MILIP silencing induced apoptosis as manifested by cleavage of caspase-3 or -7, and PARP (a) and propidium iodide (PI) / Annexin V staining assays (b). Data are representatives or mean ± s.d.; n = 3 independent experiments, One-way ANOVA followed by Tukey's multiple comparisons test. **c,** Induced knockdown of MILIP caused cell cycle arrest in G0/G1 phase detected using propidium iodide staining followed by flow cytometry. Data are mean ± s.d.; n = 3 independent experiments, two-tailed Student's *t*-test. **d,** MILIP silencing reduced A549 cell clonogenicity, which was attenuated by co-silencing of p53 siRNA2. Scale bar, 1 cm. Data are representatives or mean ± s.d.; n = 3 independent experiments, One-way ANOVA followed by Tukey's multiple comparisons test. **e,** Inhibition of MILIP transcription by sgRNA-mediated CRISPR interference reduced clonogenicity in A549 cells. Data shown represent three independent experiments. Source data of Supplementary Figs. 7a-d are provided as a Source Data file.

Supplementary Figure 8

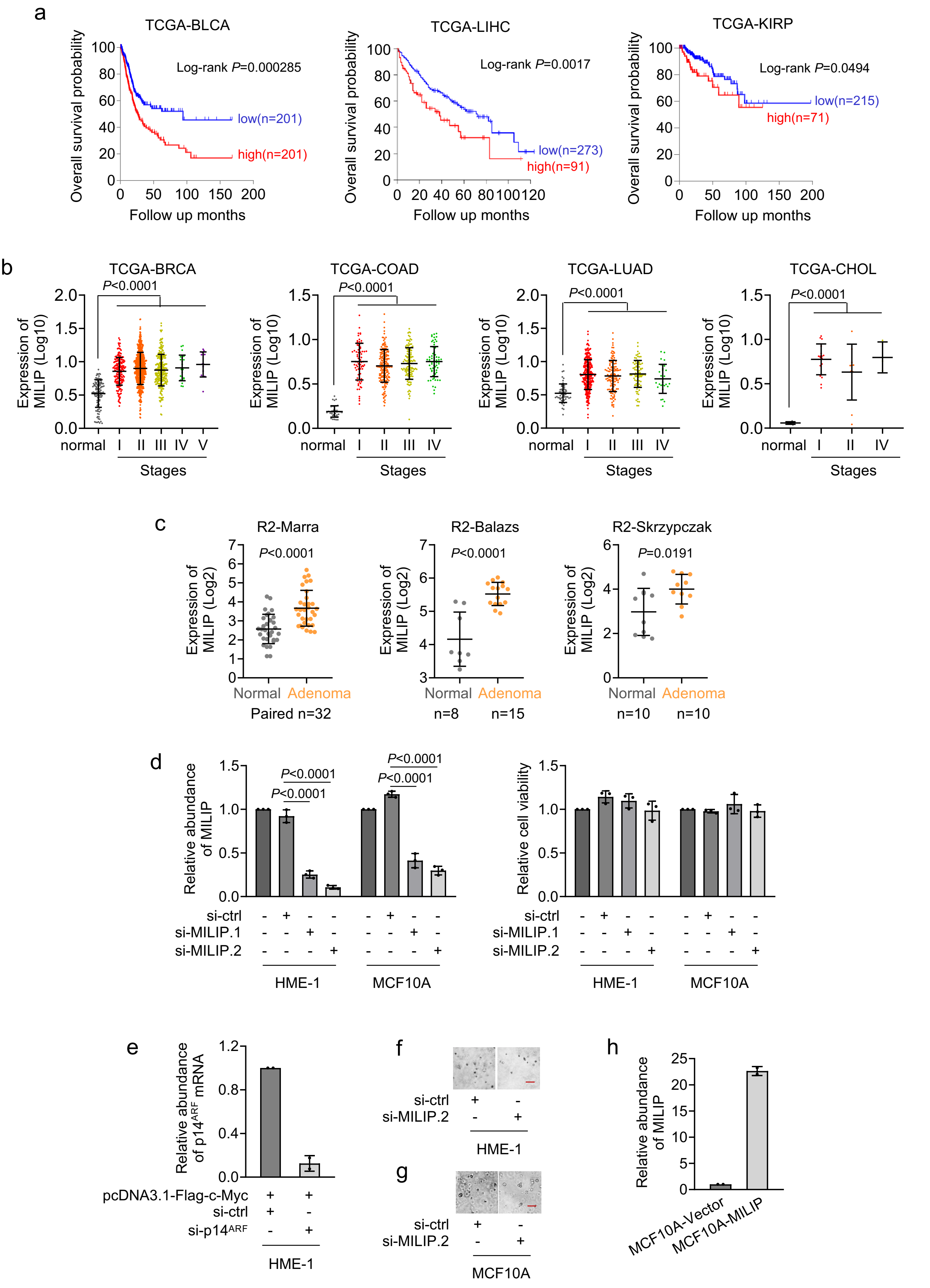

Supplementary Figure 8. MILIP expression is negatively associated with patient survival and occurs at early stages during tumour development

a, Kaplan-Meier analysis of the probability of overall survival (OS) of bladder urothelial carcinoma (BLCA; n=402 biologically independent samples), liver hepatocellular carcinoma (LIHC; n = 364 biologically independent samples), and kidney renal papillary cell carcinoma (KIRP; n = 283 biologically independent samples), in cancer datasets derived from the TCGA and the R2 Genomics Analysis and Visualization Platform (R2). **b,** MILIP levels did not differ among tumours of different stages in cancer datasets derived from the TCGA. Data are mean ± s.d.; One-way ANOVA followed by Tukey's multiple comparisons test. **c,** MILIP levels were upregulated in colon adenomas compared with normal colon epithelia in datasets derived from the R2 Genomics Analysis and Visualization Platform (R2). Data are mean ± s.d.; two-tailed Student's *t*-test. **d,** MILIP knockdown (left) did not influence the viability (right) of HME-1 and MCF10A human mammary epithelial cells. Data are mean ± s.d.; n = 3 independent experiments, One-way ANOVA followed by Tukey's multiple comparisons test. **e,** Knockdown efficiency of $p14^{ARF}$ by siRNA in HME-1 cells. Data are mean ± s.d.; n = 2 independent experiments. **f, g,** MILIP silencing itself did not induce anchorage-independent growth of HME-1 (f) and MCF10A (g) human mammary epithelial cells. Scale bar, 50 µm. Data shown represent three independent experiments. **h,** Stable overexpression efficiency of MILIP in MCF10A cells. Data are mean ± s.d.; n = 2 independent experiments. Source data of Supplementary Figs. 8a-e, h are provided as a Source Data file.

Supplementary Figure 9

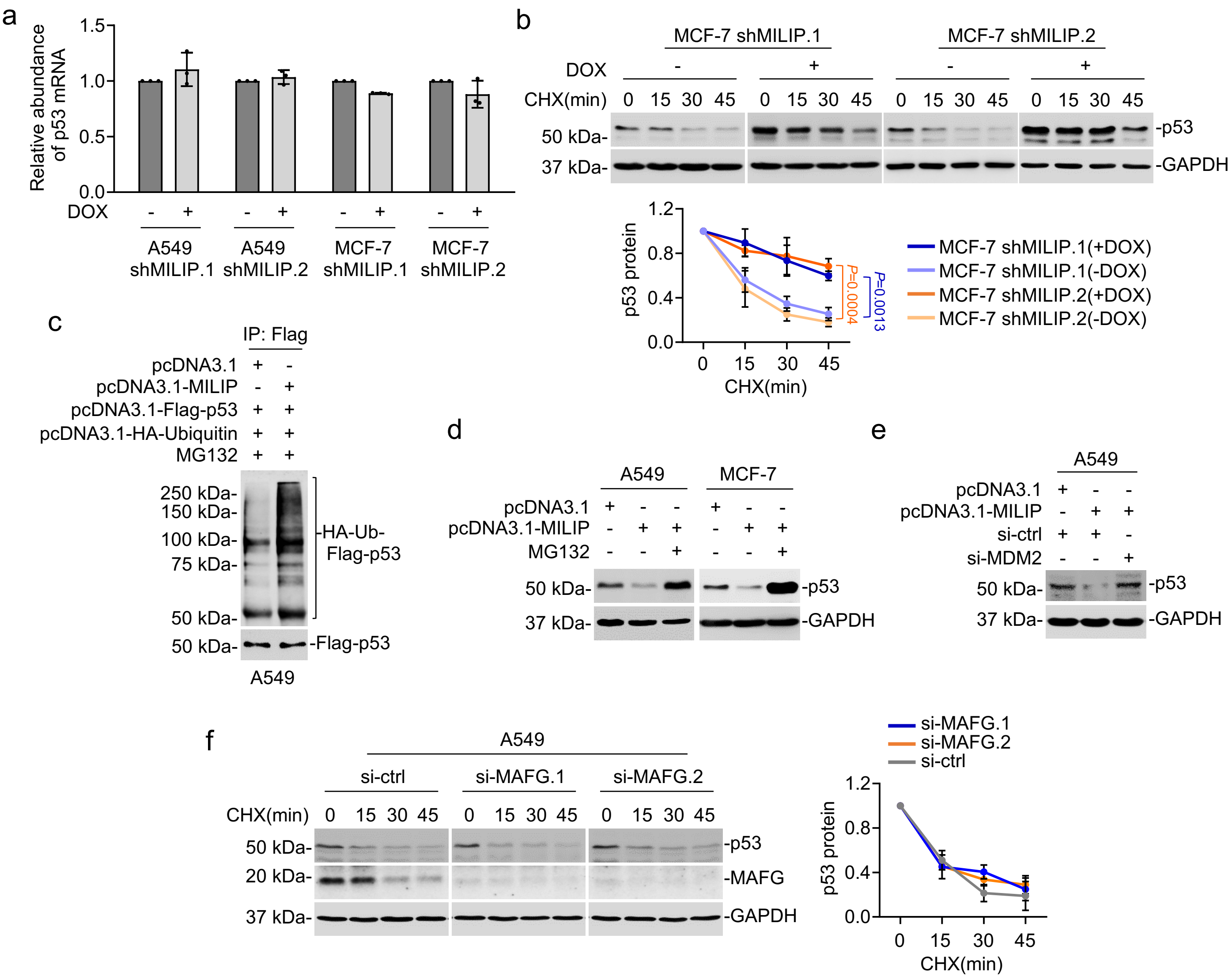

Supplementary Figure 9. MILIP promotes MDM2-mediated p53 polyubiquitination and degradation

a, Induced knockdown of MILIP did not significantly influence the expression of p53 mRNA detected using qPCR. Data are mean ± s.d.; n = 3 independent experiments. **b,** Induced knockdown of MILIP prolonged the half-life time of p53 protein in CHX-chase assays in MCF-7 cells. Data are representatives or mean ± s.d.; n = 3 independent experiments, two-tailed Student's *t*-test. CHX: 40 µg/ml, DOX: 200 ng/ml. **c,** Overexpression of MILIP promoted p53 polyubiquitination. Data shown represent three independent experiments. MG132: 10 µM. **d,** MILIP overexpression downregulated p53 expression, which was reversed by treatment with MG-132 (10 µM). Data shown represent three independent experiments. **e,** Knockdown of MDM2 diminished the decrease in p53 expression caused by MILIP overexpression. Data shown represent three independent experiments. **f,** Knockdown of MAFG did not influence the half-life time of p53 protein in CHX-chase assays in A549 cells. Data are representatives or mean ± s.d.; n = 3 independent experiments. CHX: 40 µg/ml. Source data of Supplementary Figs. 9a-f are provided as a Source Data file.

Supplementary Figure 10

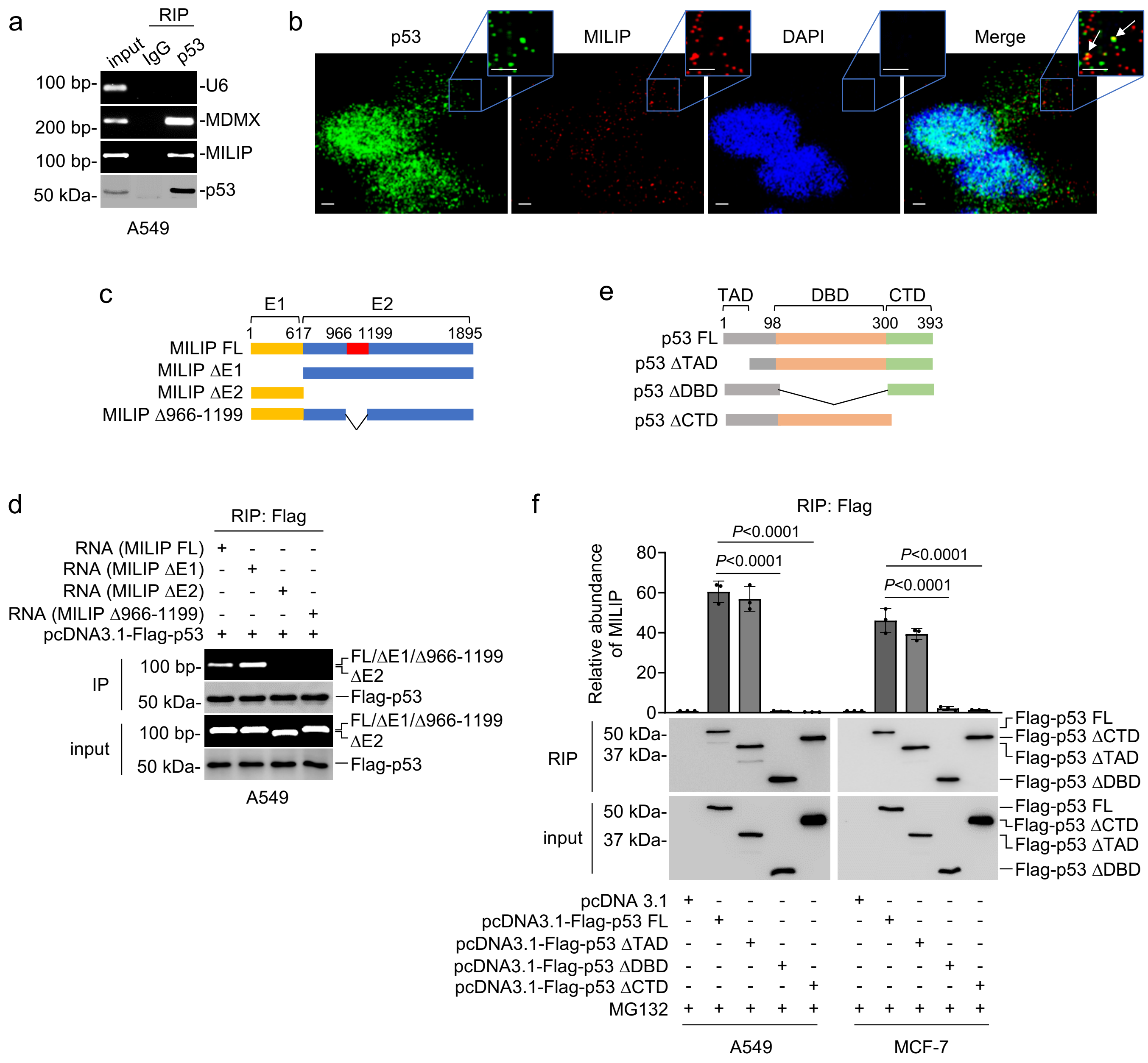

Supplementary Figure 10. p53 DBD and CTD domains and MILIP -966/-1199 fragments are necessary for their interaction

a, Co-precipitation of MILIP and p53 was detected using RIP assays. U6 RNA was used as a negative control, whereas MDMX mRNA was used as a positive control. Data shown represent three independent experiments. **b,** Co-localization of MILIP and p53 was identified using fluorescence *in situ* hybridization (FISH) analysis of MILIP in conjunction with immunofluorescent staining of p53. Data shown represent three independent experiments. Scale bar, 2 µm. **c,** A schematic illustration of full-length (FL) MILIP and MILIP deletion mutants used. MILIP ΔE1: a MILIP mutant with its exon 1 deleted; MILIP ΔE2: a MILIP mutant with its exon 2 deleted; MILIP Δ-966/-1199: a MILIP mutant with the -966/-1199 segment within exon 2 deleted. **d,** MILIP FL and MILIP ΔE1 but not MILIP ΔE2 or MILIP Δ-966/-1199 were coprecipitated with p53 as detected using RIP assays. Data shown represent three independent experiments. **e,** A schematic illustration of full-length (FL) p53 and the corresponding p53 deletion mutants. TAD: transactivation domain; DBD: DNA-binding domain; CTD: C-terminal domain. **f,** p53 FL and p53 ΔTAD but not p53 ΔDBD or p53 ΔCTD were co-pulled down with MILIP as detected using RIP assays. Data are representatives or mean ± s.d.; n = 3 independent experiments, One-way ANOVA followed by Tukey's multiple comparisons test. Source data of Supplementary Figs. 10a, d, f are provided as a Source Data file.

Supplementary Figure 11

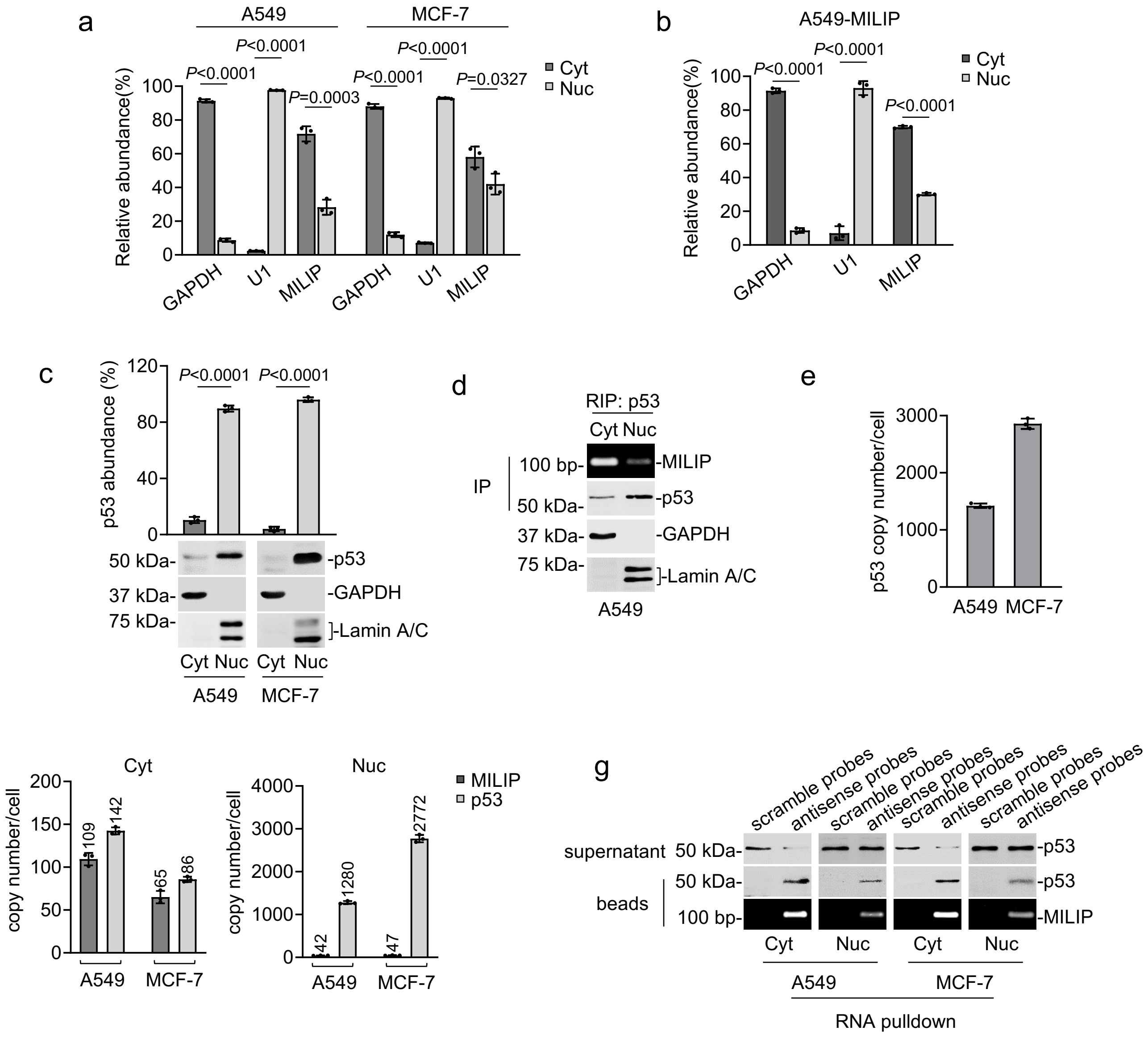

Supplementary Figure 11. Stoichiometric amounts of MILIP to interact with p53 in the cytoplasm

a,b, Both endogenous MILIP (a) and exogenous overexpressed MILIP (b) were mainly detected in cytoplasm as detected using subcellular fractionation analysis followed by qPCR. Data are mean ± s.d.; n = 3 independent experiments, two-tailed Student's *t*-test. Cyt: cytoplasm; Nuc: nucleus. **c,** p53 localised in both nucleus and cytoplasm as detected using subcellular fractionation analysis. Data are representatives or mean ± s.d.; n = 3 independent experiments, two-tailed Student's *t*-test. Cyt: cytoplasm; Nuc: nucleus. **d,** The relatively small amount of p53 in the cytoplasm was co-precipitated with a larger amount of MILIP using RIP assays. Data shown represent three independent experiments. **e,** p53 protein copy numbers were calculated based on enzyme linked immunosorbent assay (ELISA). Data are mean ± s.d.; n = 3 independent experiments. **f,** Cytoplasmic and nuclear copy numbers of MILIP and p53 were calculated based on the total copy numbers per cell and the subcellular proportion of each molecular. Data are mean ± s.d.; n = 3 independent experiments. Cyt: cytoplasm; Nuc: nucleus. **g,** p53 was approximately depleted in the cytoplasmic fractions, whereas significant amounts of p53 remained in the nuclear fractions of A549 and MCF-7 cells after MILIP pulldown. Data shown represent three independent experiments. Cyt: cytoplasm; Nuc: nucleus. Source data of Supplementary Figs. 11a-g are provided as a Source Data file.

Supplementary Figure 12

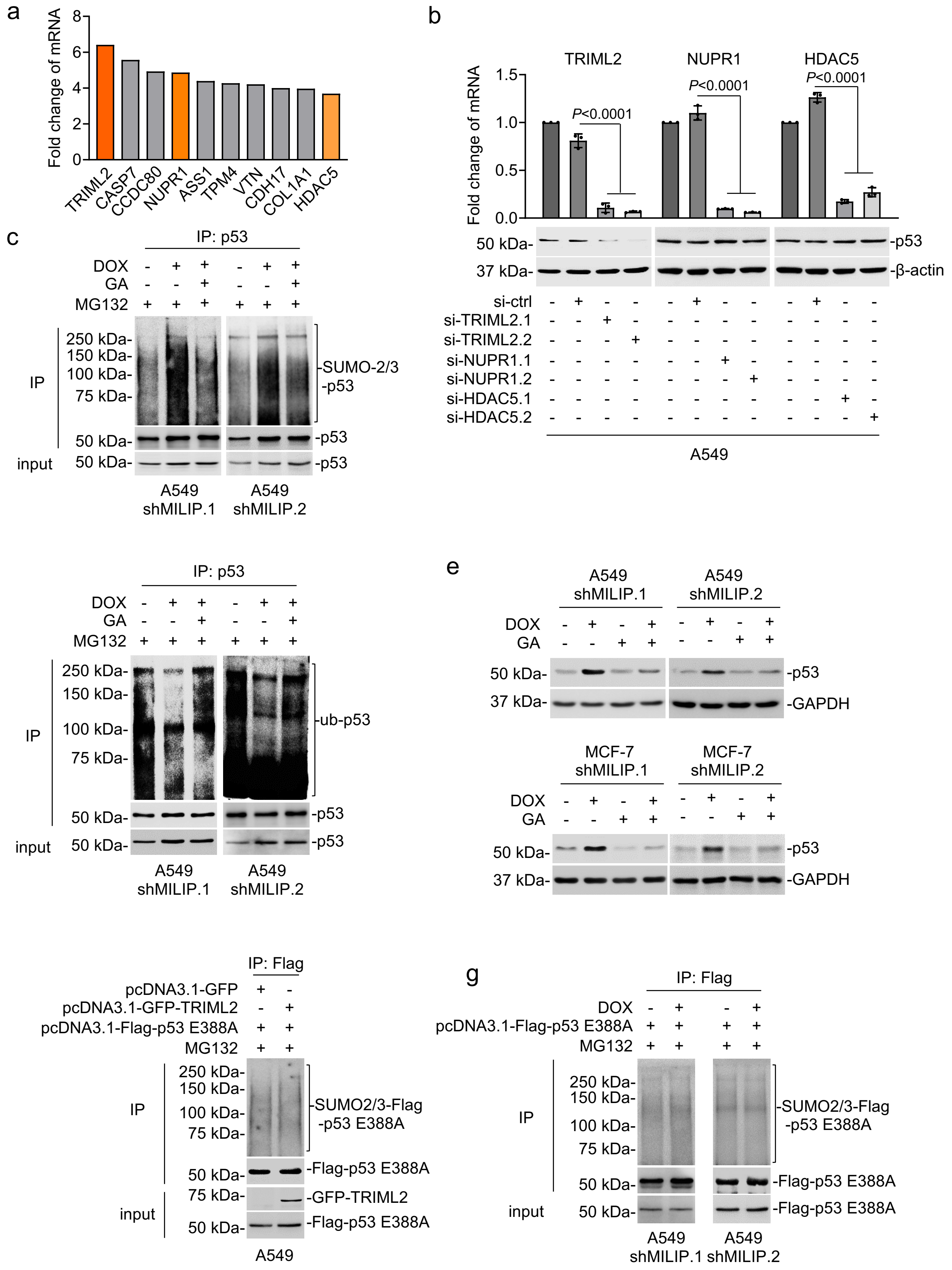

Supplementary Figure 12. MILIP supresses TRIML2-mediated p53 SUMOylation
a, p53-interacting proteins TRIML2, NUPR1 and HDAC5 are among the top 10 most upregulated genes caused by knockdown of MILIP as detected using RNA-seq. n = 1 independent experiments. **b,** Knockdown of TRIML2 but not NUPR1 and HDAC5 decreased p53 protein expression in A549 cells. Data are representatives or mean ± s.d.; n = 3 independent experiments, One-way ANOVA followed by Tukey's multiple comparisons test. CHX: 40 µg/ml. **c,** Induced knockdown of MILIP promotes p53 SUMOylation, which was attenuated by treatment with the SUMOylation inhibitor ginkgolic acid (GA). Data shown represent three independent experiments. MG132 :10 µM; GA: 20 µM, DOX: 200 ng/ml. **d,** Induced knockdown of MILIP decreased p53 polyubiquitination, which was attenuated by treatment with GA. Data shown represent three independent experiments. MG132: 10 µM; GA: 20 µM, DOX: 200 ng/ml. **e,** Induced knockdown of MILIP upregulated p53, which was abolished by treatment with GA. Data shown represent three independent experiments. GA: 20 µM, DOX: 200 ng/ml. **f,** Overexpression of TRIML2 did not alter SUMOylation of the p53 E388A mutant. Data shown represent three independent experiments. MG132: 10 µM. **g,** Knockdown of MILIP did not alter SUMOylation of the E388A p53 mutant. Data shown represent three independent experiments. MG132: 10 µM, DOX: 200 ng/ml. Source data of Supplementary Figs. 12a-g are provided as a Source Data file.

Supplementary Figure 13

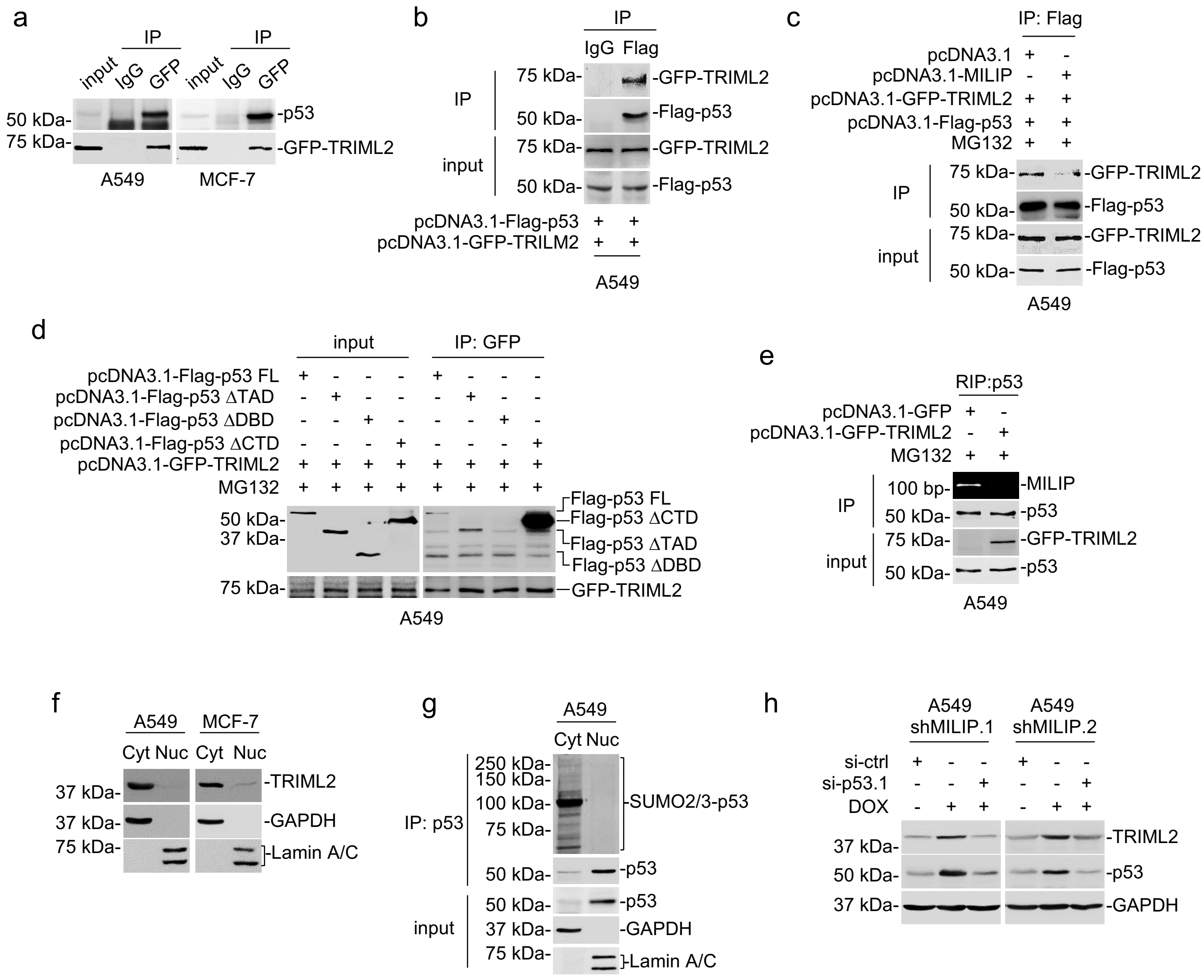

Supplementary Figure 13. TRIML2 competes with MILIP for binding to p53.
a, p53 was coimmunoprecipitated with GFP-tagged TRIML2 by an anti-GFP antibody. Data shown represent three independent experiments. IP, immunoprecipitation. **b,** GFP-tagged TRIML2 was coprecipitated with Flag-tagged p53 by an anti-Flag antibody. Data shown represent three independent experiments. **c,** Overexpression of MILIP diminished the binding between TRIML2 and p53. Data shown represent three independent experiments. MG132: 10 µM. **d,** Full-length p53 (p53 FL), the p53 mutant with the TAD deleted (p53 ΔTAD) and the p53 mutant with the CTD deleted (p53 ΔCTD) but not the p53 mutant with the DBD deleted (p53 ΔDBD) was co-pulled down with TRIML2 using immunoprecipitation assay. Data shown represent three independent experiments. MG132: 10 µM. **e,** Overexpression of TRIML2 reduced the binding between MILIP and p53 using RIP assay. Data shown represent three independent experiments. MG132: 10 µM. **f,** TRIML2 mainly localised in cytoplasm but not in nucleus as shown by subcellular fractionation analysis. Data shown represent three independent experiments. Cyt: cytoplasm; Nuc: nucleus. **g,** p53 SUMOylation was mainly detected in cytoplasm but not in nucleus using subcellular fractionation analysis followed by immunoprecipitation assay. Data shown represent three independent experiments. Cyt: cytoplasm; Nuc: nucleus. **h,** Induced knockdown of MILIP upregulated TRIML2, which was abolished by co-knockdown of p53. Data shown represent three independent experiments. DOX: 200 ng/ml. Source data of Supplementary Figs. 13a-h are provided as a Source Data file.

Supplementary Figure 14

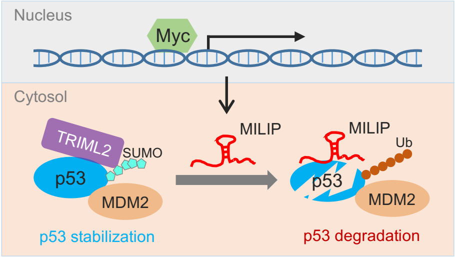

Supplementary Figure 14. c-Myc inactivation of p53 through MILIP in cancer cells
A schematic model illustrating that c-Myc inactivates p53 through transcriptionally upregulating the lncRNA MILIP that competes with TRIML2 for binding to p53, thus leading to the decrease in p53 SUMOylation and the increase in p53 polyubiquitination and subsequent degradation.

Supplementary Figure 15

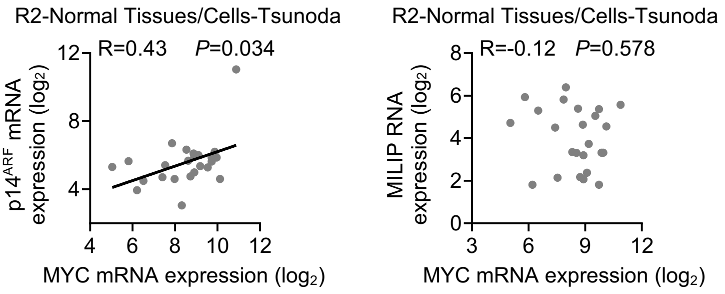

Supplementary Figure 15. MILIP is not correlated with c-Myc expression levels in normal tissues. The expression of $p14^{ARF}$ but not MILIP is correlated with c-Myc expression levels in normal tissues. Two-tailed Pearson correlation coefficient test. Source data are provided as a Source Data file.

Supplementary Figure 16

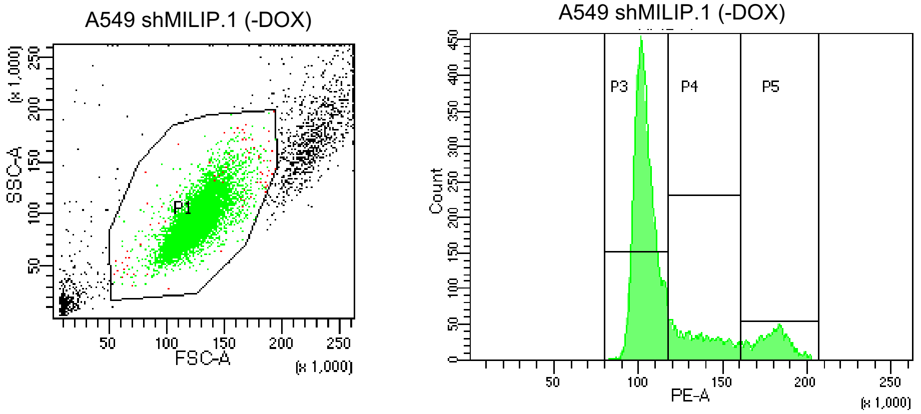

Supplementary Figure 16. Representative FACS sequential gating strategy for cell cycle analysis. (Related to Supplementary Figure 7c). Left panel demonstrates the gating strategy on a SSC vs FSC plot following capture of 10,000 events on a FACSCanto flow cytometer (BD Biosciences). This gate setting used in all subsequent sample runs. Right panel demonstrates typical cell cycle plot obtained using these gate settings.

Supplementary Tables

Supplementary Table 1. Summary of clinicopathological characteristics of the cohort of 84 colon cancer patients

Characteristics	Cases	MILIP abundance in colon cancers (RS[1])	*P* value[2]
**Gender**	84		
Male	48	33.979 ± 8.310(3)	0.643
Female	36	28.667 ± 7.162	
**Age at diagnosis**	84		
<68(4)	40	33.825 ± 8.499	0.722
≥68	44	29.773 ± 7.556	
**TNM Stage**	84		
I/II	45	44.8 ± 8.848	0.012
III/IV	39	16.590 ± 5.773	
**Histological Grade**	84		
I/II	72	32.514 ± 6.062	0.726
III	12	26.833 ± 15.767	

[1]RS: Reactive score
[2]Two-tailed Student's *t*-test; a *P* value less than 0.05 was considered statistically significant
[3]Data shown are mean ± s.e.m.
[4]The median age of the patients in this cohort was 68

Supplementary Table 2. Summary of clinicopathological characteristics of the cohort of 67 lung cancer patients

Characteristics	Cases	MILIP abundance in colon cancers (RS[1])	*P* value[2]
**Gender**	67		
Male	39	4.5 ± 0.966[(3)]	0.186
Female	28	6.812 ± 1.535	
**Age at diagnosis**	84		
<63[(4)]	33	7.485 ± 1.360	0.019
≥63	34	3.506 ± 0.959	
**TNM Stage**	84		
I/II	41	5.905 ± 1.109	0.524
III/IV	26	4.773 ± 1.367	
**Histological Grade**	84		
I/II	52	5.888 ± 1.012	0.363
III	15	4 ± 1.533	

[1]RS: Reactive score
[2]Two-tailed Student's *t*-test; a *P* value less than 0.05 was considered statistically significant
[3]Data shown are mean ± s.e.m.
[4]The median age of the patients in this cohort was 63

Supplementary Table 3. Sequence similarity of transcripts in other species compared with human MILIP

Description	Total score	Query cover	E value
Homo sapiens	4405	100%	0.0
Pan troglodytes	3804	93%	0.0
Mus musculus	44.6	2%	0.23

Supplementary Table 4. List of antibodies

Antibody (Ab)	Catalogue NO.	Company	Dilution
PARP Mouse mAb	sc-8007	Santa Cruz Biotechnology (Dallas, TX)	1:200
Caspase 3 Rabbit pAb	9662	Cell Signaling Technology (Danvers, MA)	1:500
Caspase 7 Mouse mAb	sc-28295	Santa Cruz Biotechnology (Dallas, TX)	1:200
c-Myc Rabbit mAb	5605	Cell Signaling Technology (Danvers, MA)	1:800
GAPDH Mouse mAb	sc-32233	Santa Cruz Biotechnology (Dallas, TX)	1:2000
c-Myc Rabbit pAb	9402	Cell Signaling Technology (Danvers, MA)	1:50
p21 Mouse mAb	05-345	Sigma-Aldrich (Saint Louis, USA)	1:500
p27 Mouse mAb	sc-1641	Santa Cruz Biotechnology (Dallas, TX)	1:500
Flag Mouse mAb	F3165	Sigma-Aldrich (Saint Louis, MO)	1:1000
Ubiquitin Mouse mAb	3936S	Cell Signaling Technology (Danvers, MA)	1:800
TRIML2 Rabbit pAb	ab87292	Abcam (Cambridge, UK)	1:1000
MAFG Rabbit pAb	Ab154318	Abcam (Cambridge, UK)	1:1000
Puma Rabbit pAb	4976	Cell Signaling Technology (Danvers, MA)	1:1000
PERP Rabbit pAb	ab5986	Abcam (Cambridge, UK)	1:1000
p53 Mouse mAb	sc-126	Santa Cruz Biotechnology (Dallas, TX)	1:1000
MDM2 Rabbit mAb	86934	Cell Signaling Technology (Danvers, MA)	1:800
HA Mouse mAb	2367	Cell Signaling Technology (Danvers, MA)	1:1000
SUMO2/3 Rabbit mAb	4971	Cell Signaling Technology (Danvers, MA)	1:800
GFP Mouse mAb	A-11120	Thermo Fisher Scientific (Waltham, MA)	1:200
Beta Actin Mouse mAb	66009	Proteintech Group (Wuhan, Hubei, P.R.C)	1:10000
Lamin A/C Rabbit pAb	2032	Cell Signaling Technology (Danvers, MA)	1:1000
Mouse TrueBlot® ULTRA: Anti-Mouse Ig HRP mAb	18-8817-33	Rockland Immunochemicals (Limerick, PA)	1:1000
Rabbit TrueBlot®: Anti-Rabbit IgG HRP mAb	18-8816-31	Rockland Immunochemicals (Limerick, PA)	1:1000
Normal mouse IgG	sc-2025	Santa Cruz Biotechnology (Dallas, TX)	1: 500
Goat Anti-Mouse IgG(H+L)-HRP Conjugate	1706516	Bio-Rad Laboratories (Hercules, CA)	1:2500
Goat Anti-Rabbit IgG (H+L)-HRP Conjugate	1706515	Bio-Rad Laboratories (Hercules, CA)	1:2500

mAb: monoclonal antibody
pAb: polyclonal antibody

Supplementary Table 5. List of qRT-PCR primers

MILIP	Forward: AGAACCGCGAAAGGCTACTG<br>Reverse: CACTTAAAGCCGGTCGTGGA
p53	Forward: GGAAATTTGCGTGTGGAGTATTT<br>Reverse: GTTGTAGTGGATGGTGGTACAG
TRIML2	Forward: GACCGAGATGTCCCTCATTTAC<br>Reverse: GTCTGGACTTGTGTCTCCATTT
PUMA	Forward: TGGAGGGTCCTGTACAATCT<br>Reverse: CACCTAATTGGGCTCCATCTC
PERP	Forward: TGTCAGAGCCTCATGGAGTA<br>Reverse: GCGAAGAAGGAGAGGATGAAA
p21	Forward: CCAGCCTCTGGCATTAGAATTA<br>Reverse: CGGGATGAGGAGGCTTTAAATA
NUPR1	Forward: GGTCGCACCAAGAGAGAAGC<br>Reverse: CTCCGCAGTCCCGTCTCTAT
HDAC5	Forward: TTCTTTGGACCAGAGTTCCC<br>Reverse: GTTGGGTTCAGAGGCTGTTT
GAPDH	Forward: CTCCTCCTGTTCGACAGTCA<br>Reverse: CAATACGACCAAATCCGTTG
18s rRNA	Forward: GCTTAATTTGACTCAACACGGGA<br>Reverse: AGCTATCAATCTGTCAATCCTGTC
U1	Forward: GGCGAGGCTTATCCATTG<br>Reverse: CCCACTACCACAAATTATGC

Supplementary Table 6. shRNA, siRNA and sgRNA sequences

MILIP	siRNA1/shRNA.1: GGAGTCAGGGCAATTCCAA<br>SiRNA2/shRNA.2: GGTAACATAGAGACCCTAT
MILIP-CRISPRi sgRNA	sgRNA: CGCGGCCGGCCGGCGCTTAA
TRIML2	shRNA.1: GCAAGAAAGAATGGCGATGAT<br>shRNA.2: GGCAACCCAGAAAGATTGGATTTC
c-Myc	siRNA.1: CCUGAGACAGAUCAGCAACAAC<br>siRNA.2: GGACUAUCCUGCUGCCAAG
p53	siRNA.1: CGGCGCACAGAGGAAGAGAAUCUC<br>siRNA.2: GACUCCAGUGGUAAUCUAC
MDM2	siRNA: GGCCAGUAUAUUAUGACUA
MAX	siRNA.1: GCCACAGAAUAUAUCCAGU<br>siRNA.2: GAGCAACCGAGGUUUCAAU
MAFG	siRNA.1: GCGUCAGAGCUCAAGGAAU<br>siRNA.2: GCGUUAGUUGGAAUCAUAU
NUPR1	siRNA.1: GGAUGAAUCUGACCUCUAU<br>siRNA.2: GCAGAAUUCAGAGAGGAAG
HDAC5	siRNA.1: CCUCCUACAAACUGCCUUU<br>siRNA.2: GCUAUGACAACGGGAACUU
c-Myc-BR sgRNA	sgRNA1: TCACATGATGTTTGGTCACG<br>sgRNA2: CCGCGTCGCCGCTTCATGAA

Supplementary Table 7. List of RT-PCR primers, RNA pulldown probes and FISH probes

ChIP-c-Myc-BR	Forward: ATGATGTTTGGTCACGTGGGCTCCATT<br>Reverse: TTGCGCATGCGCAGTTTGCCCT
c-Myc-BR	Forward: CCGCACTCACCACACTG<br>Reverse: TGCCCTAGGCTGAGCAC
MILIP ΔE1	Forward: AGACCCAAGCTGGATGAGAGCTGAAGGTGTTC<br>Reverse: GAACACCTTCAGCTCTCATCCAGCTTGGGTCT
MILIP Δ 966-1199	Forward: TCACAGCTGGATGCCTCCTACCTTAAGACCCC<br>Reverse: GGGGTCTTAAGGTAGGAGGCATCCAGCTGTGA
p53 E388A	Forward: GTCTGAGTCAGGCCCTGCTGTCTTGAACATGAG<br>Reverse: CTCATGTTCAAGACAGCAGGGCCTGACTCAGAC
U6	Forward: TGGAACGCTTCACGAATTTGCG<br>Reverse: GGAACGATACAGAGAAGATTAGC
MDMX	Forward: CAGCAGGTGCGCAAGGTGAA<br>Reverse: CTGTGCGAGAGCGAGAGTCTG
MILIP-Biotin-AS-probes	Antisense 1: TGACCACGGAACACCTTCAG<br>Antisense 2: ACTGTGTCCAGGGACAAGTG<br>Antisense 3: TGAGAGGGATGCTTGGAAACC
MILIP-Biotin-S-probes	Scramble 1: GACAATACTCGACAGGCTCC<br>Scramble 2: GAGCGAAGGTTATGTCGACC<br>Scramble 3: GTGAACAACGCGAGTTGTGGA
MILIP FISH/ISH probes	Antisense A: GAAAGTCCAGACACGGGATCC<br>Antisense B: GGTTGGAATTGCCCTGACTCC<br>Antisense C: GTTTCTTGGTTGGAATTGCCC<br>Antisense D: TGACCACGGAACACCTTCAG<br>Antisense E: ACTGTGTCCAGGGACAAGTG<br>Antisense F: TGAGAGGGATGCTTGGAAACC
