# Supplementary material for: c-Myc inactivation of p53 through the pan-cancer lncRNA MILIP drives cancer pathogenesis
Source: Nat Commun. 2020 Oct 5;11:4980. doi: 10.1038/s41467-020-18735-8 (PMC7536215; doi:10.1038/s41467-020-18735-8)
Supplement: Supplementary file 3 — Reporting Summary [file 41467_2020_18735_MOESM3_ESM.pdf]

nature research

Corresponding author(s): Lei Jin

Last updated by author(s): Aug 26, 2020

Reporting Summary

Nature Research wishes to improve the reproducibility of the work that we publish. This form provides structure for consistency and transparency in reporting. For further information on Nature Research policies, see Authors & Referees and the Editorial Policy Checklist.

Statistics

For all statistical analyses, confirm that the following items are present in the figure legend, table legend, main text, or Methods section.

n/a	Confirmed	
☐	☒	The exact sample size (*n*) for each experimental group/condition, given as a discrete number and unit of measurement
☐	☒	A statement on whether measurements were taken from distinct samples or whether the same sample was measured repeatedly
☐	☒	The statistical test(s) used AND whether they are one- or two-sided<br>*Only common tests should be described solely by name; describe more complex techniques in the Methods section.*
☒	☐	A description of all covariates tested
☐	☒	A description of any assumptions or corrections, such as tests of normality and adjustment for multiple comparisons
☐	☒	A full description of the statistical parameters including central tendency (e.g. means) or other basic estimates (e.g. regression coefficient) AND variation (e.g. standard deviation) or associated estimates of uncertainty (e.g. confidence intervals)
☐	☒	For null hypothesis testing, the test statistic (e.g. *F*, *t*, *r*) with confidence intervals, effect sizes, degrees of freedom and *P* value noted<br>*Give P values as exact values whenever suitable.*
☒	☐	For Bayesian analysis, information on the choice of priors and Markov chain Monte Carlo settings
☒	☐	For hierarchical and complex designs, identification of the appropriate level for tests and full reporting of outcomes
☐	☒	Estimates of effect sizes (e.g. Cohen's *d*, Pearson's *r*), indicating how they were calculated

Our web collection on statistics for biologists contains articles on many of the points above.

Software and code

Policy information about availability of computer code

Data collection	BD FACSDiva V8.0.1 software; LAS-4000 Image Reader V2.1 software; SDS V2.4 software; AxioVision V4.7.1.0 software; Image lab V5.2.1 software; Gen5 V1.04.5 software; LAS X V3.7.2.22383 software.
Data analysis	Microsoft Excel 2016 software; GraphPad Prism V8.4.2 software; adobe photoshop elements V12.0 software; ImageJ V1.50e software; Ingenuity Pathway Analysis (IPA) V52912811 software

For manuscripts utilizing custom algorithms or software that are central to the research but not yet described in published literature, software must be made available to editors/reviewers. We strongly encourage code deposition in a community repository (e.g. GitHub). See the Nature Research guidelines for submitting code & software for further information.

Data

Policy information about availability of data

All manuscripts must include a data availability statement. This statement should provide the following information, where applicable:

- Accession codes, unique identifiers, or web links for publicly available datasets
- A list of figures that have associated raw data
- A description of any restrictions on data availability

The RNA sequencing data have been deposited in the NCBI Gene Expression Omnibus database under the accession code GSE141886 (https://www.ncbi.nlm.nih.gov/geo/query/acc.cgi?acc=GSE141886). The long noncoding RNA expression data referenced during the study are available in a public repository from the Cancer RNA-seq Nexus dataset (http://syslab4.nchu.edu.tw/). MILIP expression data in adenoma and normal tissues referenced during the study are available in a public repository from the R2 website (https://hgserver1.amc.nl/cgi-bin/r2/main.cgi) under the accession codes Mixed Colon-Marra (GSE8671), Mixed Colon-Balazs (GSE4183), and Mixed Colon-Skrzypczak (GSE20916). The linear regression data referenced during the study are available in a public repository from the R2 website (https://hgserver1.amc.nl/cgi-bin/r2/main.cgi) under the accession codes TCGA-Glioblastoma, Disease Colon-Watanabe (GSE3629), Tumor Breast-Black (GSE36771), and Normal Tissues/Cells-Tsunoda (GSE18674). The cancer patient survival data referenced during the study are available in a public repository from the GEPIA website (http://gepia.cancer-pku.cn/) and the OncoLnc website (http://www.oncolnc.org/) under the accession codes TCGA-LUAD, TCGA-BRCA, TCGA-BLCA, TCGA-LIHC, TCGA-KIRP. The source data underlying Figs. 1a-c, e-h, 2a-h, 3a-g, 4a-h, 5a-f, and Supplementary Figs. 1b, 2b-h, j-m, 3a, b, d,

4b-g, i, 5a-n, 6a, b, e-g, 7a-d, 8a-e, h, 9a-f, 10a, d, f, 11a-g, 12a-g, 13a-h, 15 are provided as a Source Data file. All the other data supporting the findings of this study are available within the article and its supplementary information files and from the corresponding author upon reasonable request.

Field-specific reporting

Please select the one below that is the best fit for your research. If you are not sure, read the appropriate sections before making your selection.

☒ Life sciences ☐ Behavioural & social sciences ☐ Ecological, evolutionary & environmental sciences

For a reference copy of the document with all sections, see nature.com/documents/nr-reporting-summary-flat.pdf

Life sciences study design

All studies must disclose on these points even when the disclosure is negative.

Sample size	No study size calculation was performed and the sample size was based on our prior studies using the same types of assays and published literature to ensure statistically significant results. All key experiments were repeated independently using different cell lines or different techniques. For in vitro studies n=3 was used as a standard sample size. For in vivo mouse model, n=6 was chosen to detect the difference of tumor size and weight among different groups. For human tissue samples, sample size was determined by the availability of samples.
Data exclusions	No data were excluded for this study.
Replication	n=3 biological replicates for in vitro cellular experiments unless otherwise specified. The in vivo animal experiments were performed with 6 mice per group.
Randomization	The cells and animals were randomly grouped for the experiments. Randomization was not relevant for human tissue samples as the samples were grouped according to their pathology (normal, carcinoma and adenoma).
Blinding	For experiments using cell lines the investigators were not blinded during data acquisition and analysis. The application of treatments and processing procedures negated the possibility of blinding but there was no human bias given all data was collected independently using instrumentation. Similarly, in the animal experiments the investigator was not blinded to the group allocation as the same investigator both planned and performed the experiment. Two observers measured volumes/weights to alleviate human bias in these data. The scoring and quantification of human tissue samples were performed in a blinded manner.

Reporting for specific materials, systems and methods

We require information from authors about some types of materials, experimental systems and methods used in many studies. Here, indicate whether each material, system or method listed is relevant to your study. If you are not sure if a list item applies to your research, read the appropriate section before selecting a response.

Materials & experimental systems

n/a	Involved in the study	
☐	☒	Antibodies
☐	☒	Eukaryotic cell lines
☒	☐	Palaeontology
☐	☒	Animals and other organisms
☐	☒	Human research participants
☒	☐	Clinical data

Methods

n/a	Involved in the study	
☒	☐	ChIP-seq
☐	☒	Flow cytometry
☒	☐	MRI-based neuroimaging

Antibodies

Antibodies used	All antibodies and relative information about their species, catalog numbers, companies and dilution have been mentioned in the method section. PARP (sc-8007), Caspase 7 (sc-28295), GAPDH (sc-32233), p27 (sc-1641), p53 (sc-126) and normal mouse IgG (sc-2025) were purchased from Santa Cruz Biotechnology; Caspase 3 (9665), c-Myc (5605, 9402), Ubiquitin (3936S), Puma (4976), MDM2 (86934), HA (2367), SUMO2/3 (4971) and Lamin A/C (2032) were purchased from Cell Signaling Technology; p21 (05-345) and Flag (F3165) were from Sigma-Aldrich; TRIML2 (ab87292), MAFG (ab154318) and PERP (ab5986) were from Abcam; GFP (A-11120) was from Thermo Fisher Scientific; Beta actin (66009) was from Proteintech Group; Mouse TrueBlot® ULTRA (18-8817-33) and Rabbit TrueBlot (18-8816-31) were from Rockland Immunochemicals. Goat Anti-Mouse IgG(H+L)-HRP Conjugate (1706516) and Goat Anti-Rabbit IgG (H+L)-HRP Conjugate (1706515) were from Bio-Rad Laboratories.
Validation	PARP: Santa Cruz Biotechnology , Cat No. sc-8007. Santa Cruz Biotechnology website antibody validation: 1) western blot analysis of PARP expression in nuclear extracts from Ramos and K-562 cells and whole cell lysates from HL-60, Daudi and NTERA-2 cl.D1 cells shows a single band of full-length PARP at the expected MW of 116kDa and a single band of cleaved PARP at the expected MW of ~89kDa; 2) Western blot analysis of PARP expression in non-transfected control and PARP-1 siRNA transfected HeLa cells shows the absence of signal in the PARP knockdown HeLa cells of a single band at the expected MW of 116kDa, confirming the specificity of the antibody for PARP.

Caspase 3: Cell Signaling Technology, Cat No. 9662. Cell Signaling Technology website antibody validation: 1) Western blot analysis of extracts from Jurkat cells, untreated or etoposide-treated (25uM, 5hrs), and NIH/3T3 cells, untreated or staurosporine-treated (1uM, 3hrs), shows a single band of full-length caspase 3 at expected MW of 35kDa in untreated cells and both full-length and cleaved (17kDa) capase 3 signals in treated cells; 2) Western blot analysis of extracts from HCT116 cells or caspase 3 knock-out HCT116 cells shows the absence of signal in the caspase 3 knock-out cells of a single band at the expected MW of 35kDa, confirming the specificity of the antibody for caspase 3. Certificate of analysis from CST website: https://media.cellsignal.com/coa/9662/19/9662-lot-19-coa.pdf.
Caspase 7: Santa Cruz Biotechnology, Cat No. sc-28295. Santa Cruz Biotechnology website antibody validation: 1) Western blot analysis of caspase 7 expression in untreated and Staurosporine treated HeLa cell lysates and Jurkat whole cell lysate shows a single band of full-length caspase 7 at the expected MW of 28~38kDa in untreated HeLa cell lysate and Jurkat whole cell lysate and both full-length and cleaved (20kDa) caspase 7 signals in treated HeLa cell lysate; 2) Western blot analysis of caspase 7 expression in non-transfected 293T cells and human caspase 7 transfected HEK 293T cells shows the increased signal in caspase 7 transfected HEK 293T cells of a single band at the expected MW of 28~38kDa.
c-Myc: Cell Signaling Technology, Cat No. 5605. Cell Signaling Technology website antibody validation: 1) Western blot analysis of extracts from Jurkat, RPMI 8226 and A20 cell lines shows bands of the expected MW of 57~65kDa. 2) Western blot analysis of extracts from control HEK 293 cells or c-Myc knockout HEK 293 cells shows the absence of signal in the c-Myc knock-out cells at the expected MW of 57~65kDa. 3) Western blot analysis of extracts from HeLa cells, mock transfected or transfected with SignalSilence® c-Myc siRNA shows the absence of signal in the c-Myc siRNA transfected HeLa cells at the expected MW of 57~65kDa. Both 2)&3) confirm the specificity of the antibody for c-Myc. Certificate of analysis from CST website: https://media.cellsignal.com/ coa/5605/15/5605-lot-15-coa.pdf.
GAPDH: Santa Cruz Biotechnology , Cat No. sc-32233. Santa Cruz Biotechnology website antibody validation: 1) Western blot analysis of GAPDH expression in HepG2, A549 and Raji whole cell lysates shows a single band at the expected MW of 37kDa. 2) Western blot analysis of GAPDH expression in non-transfected HEK 293T cells and human GAPDH transfected HEK 293T cells shows the increased signal in GAPDH transfected HEK 293T cells, confirming the specificity of the antibody for GAPDH.
c-Myc: Cell Signaling Technology, Cat No. 9402. Cell Signaling Technology website antibody validation: 1) Western blot analysis of extracts from HeLa, BaF3 and NBT-11 cells show bands at the expected MW of 57~70kDa. 2) Western blot analysis of extracts from HeLa cells 48 hours following mock transfection, transfection with nonspecific (control) siRNA or transfection with c-Myc siRNA, shows decreased signal in the c-Myc siRNA transfected HeLa cells at the expected MW of 57~70kDa, confirming the specificity of the antibody for c-Myc. 3) Chromatin immunoprecipitations (ChIP) were performed with cross-linked chromatin from Daudi cells and either c-Myc or Normal Rabbit IgG antibodies. The enriched DNA was quantified by real-time PCR using human ATF4 promoter primers, human NPM1 intron 1 primers, and human α Satellite Repeat primers. The abundant signals were shown in groups using c-Myc antibody compared with IgG antibody, confirming the application of the c-Myc antibody for ChIP assay. Certificate of analysis from CST website: https://media.cellsignal.com/coa/9402/11/9402-lot-11-coa.pdf.
p21: Sigma-Aldrich, Cat No. 05-345. Sigma-Aldrich website antibody validation: Western blot analysis using 0.5-2 μg/mL of this antibody detected p21 in 20 μg of HeLa nuclear extract shows a single band at the expected MW of 21kDa. Certificate of analysis from Sigma-Aldrich website: https://www.sigmaaldrich.com/catalog/product/mm/05345?lang=en&region=AU.
p27: Santa Cruz Biotechnology, Cat No. sc-1641. Santa Cruz Biotechnology website antibody validation: 1) Western blot analysis of p27 expression in MM-142, RAW 264.7, NAMALWA, BJAB, Raji and C6 whole cell lysates show bands at the expected MW of 27kDa; 2) Western blot analysis of p27 expression in empty vector control and p27 CRISPR/CAS9 KO plasmid transfected NIH/3T3 whole cell lysates shows the decreased signal in p27 CRISPR/CAS9 KO plasmid transfected NIH/3T3 whole cell lysates at the expected MW of 27kDa; 3) Western blot analysis of p27 expression in non-transfected and human p27 transfected 293 whole cell lysates shows the increased signal in p27 transfected HEK 293 whole cell lysate at the expected MW of 27kDa. Both 2)&3) confirm the specificity of the antibody for p27.
Flag: Sigma-Aldrich, Cat No. F3165. A peptide DYKDDDDK is used as the immunogen. The Flag antibody recognizes the Flag sequence at the N-terminus, Met-N-terminus, C-terminus, or at an internal site of Flag fusion proteins. The western blot analysis of A549 cell transfected with plasmid expressing Flag-p53 protein shows a single band at the expected MW of ~53kDa (see Figure 3g right panel of manuscript).
Ubiquitin: Cell Signaling Technology, Cat No. 3936. Cell Signaling Technology website antibody validation: Western blot analysis of ubiquitin expression in HEK 293 and HeLa cells, untreated or treated with the 26S proteasome inhibitor MG132, shows increased signals in MG132 treated cells. Certificate of analysis from CST website: https://media.cellsignal.com/coa/3936/17/3936-lot-17-coa.pdf.
TRIML2: Abcam, Cat No. ab87292. Abcam website antibody validation: Western blot analysis of TRIML2 expression in human placenta lysate shows a single band at the expected MW of ~44kDa.
MAFG: Abcam, Cat No. ab154318. Abcam website antibody validation: Western blot analysis of MAFG expression in HeLa cell lysate shows a single band at the expected MW of 18kDa.
Puma: Cell Signaling Technology, Cat No. 4976. Cell Signaling Technology website antibody validation: Western blot analysis of Puma expression in extracts from RL7, HL-60 and SR cells shows a single band at the expected MW of 23kDa. Certificate of analysis from CST website: https://media.cellsignal.com/coa/4976/7/4976-lot-7-coa.pdf.
PERP: Abcam, Cat No. ab5986. Abcam website antibody validation: 1) Western blot analysis of PERP expression in extracts from 293, A549, HeLa, HepG2 and MCF-7 cells shows a single band at the expected MW of 21kDa; 2) Western blot analysis of PERP expression in A431 whole cell lysates in the absence and presence of blocking peptide shows the absence of signal in blocking peptide presenting group at the expected MW of 21kDa, confirming the specificity of the antibody for PERP.
p53: Santa Cruz Biotechnology, Cat No. sc-126. Santa Cruz Biotechnology website antibody validation: 1) Western blot analysis of p53 expression in extracts from A549, A-431, Daudi, T-47D and NTERA-2 cl.D1 cells shows a single band at the expected MW of 53kDa. 2) Western blot analysis of p53 expression in control non-transfected and p53 siRNA transfected HeLa cells shows the decreased signal in p53 siRNA transfected HeLa cells at the expected MW of 53kDa. 3) Western blot analysis of p53 expression in untreated and Actinomycin D treated MOLT-4 whole cell lysates shows the increased signal in Actinomycin D treated MOLT-4 whole cell lysate at the expected MW of 53kDa. Both 2)&3) confirm the specificity of the antibody for p53.
MDM2: Cell Signaling Technology, Cat No. 86934. Cell Signaling Technology website antibody validation: Western blot analysis of MDM2 expression in SJSA-1, U2OS, and Saos-2 cells, untreated (-) or treated with Nutlin 3a (10 μM, 24 hr; +), shows the increased signal in Nutlin 3a treated cells at the expected MW of 90kDa. Certificate of analysis from CST website: https://media.cellsignal.com/coa/86934/2/86934-lot-2-coa.pdf.
HA: Cell Signaling Technology, Cat No. 2367. Cell Signaling Technology website antibody validation: 1) Western blot analysis of extracts from COS cells, untransfected or expressing HA-tagged Akt3 or HA-tagged Estrogen Receptor (ER), shows a single band at the expected MW of 60kDa for HA-tagged Akt3 and 66kDa for HA-tagged Estrogen Receptor. 2) Flow cytometric analysis of

COS cells, untransfected or transfected with HA-Akt3 compared to a nonspecific negative control antibody, shows increased signal in COS cells transfected with HA-Akt3.
SUMO2/3: Cell Signaling Technology, Cat No. 4971. Cell Signaling Technology website antibody validation: Western blot analysis of recombinant GST-SUMO-1 protein (38kDa), recombinant SUMO-2, recombinant SUMO-3, and extracts from C6 and PC12 cells, shows a single band in recombinant SUMO-2, recombinant SUMO-3, and extracts from C6 and PC12 cells, but not in recombinant GST-SUMO-1 protein, confirming that this antibody for SUMO2/3 does not cross-react with SUMO-1. Certificate of analysis from CST website: https://media.cellsignal.com/coa/4971/7/4971-lot-7-coa.pdf.
GFP: ThermoFisher Scientific, Cat No. A-11120. ThermoFisher Scientific website antibody validation: 1) HeLa cell transfected with pShooter pCMV/myc/mito/GFP, then fixed and permeabilized. Green-fluorescent protein (GFP) localized in the mitochondria was labeled with anti-GFP mouse IgG2a and detected with orange-fluorescent Alexa Fluor® 555 goat anti-mouse IgG. 2) Western blot analysis of GFP-MON1B expression in HCT116 cell with stably expression of GFP-MON1B protein, transfected with control siRNA and MON1B siRNA, shows the decreased signal of a single band at the expected MW of 87kDa, comfirming the specificity of the antibody for GFP (Koji Yamano, et al. (2008). Elife. 7:e31326).
Beta Actin: Proteintech, Cat No. 66009. Proteintech website antibody validation: Western blot analysis of beta-actin expression in extracts from human brain tissue, HEK 293, HeLa, HepG2, PC-3, A2780, SW1990 and NIH3T3 cell lines shows a single band at the expected MW of 42kDa; 2) Western blot analysis of Beta Actin expression in control shRNA and Beta Actin shRNA transfected A549 cells shows the decreased signal in Beta Actin shRNA transfected A549 cells at the expected MW of 42kDa, confirming the specificity of the antibody for Beta Actin.
Lamin A/C: Cell Signaling Technology, Cat No. 2032. Cell Signaling Technology website antibody validation: 1) Western blot analysis of Lamin A/C expression in extracts from HeLa, Raw 264.7 and PC12 cells shows two bands at the expected MW of 70kDa. 2) Western blot analysis of Lamin A/C expression in extracts from control HeLa cells or HeLa cells with an in-frame truncation mutation in the gene encoding Lamin A/C shows the change of the MW in the mutant HeLa cells, confirming the specificity of the antibody for Lamin A/C. Certificate of analysis from CST website: https://media.cellsignal.com/coa/2032/6/2032-lot-6-coa.pdf.
Mouse TrueBlot® ULTRA: Anti-Mouse Ig HRP: Rockland Immunochemicals, Cat No. 18-8817-33. Rockland Immunochemicals website antibody validation: Mouse TrueBlot® IP / Western Blot: Caspase 7 was immunoprecipitated from 0.5 ml of 1x10e7 Jurkat cells/ml with 5 ug mouse anti-human Caspase 7, subjected to electrophoresis, transferred to an PVDF membrane, and western blotted with anti-Caspase 7 using Mouse TrueBlot® ULTRA: Anti-Mouse Ig HRP or conventional HRP-conjugated anti-mouse antibody. The heavy and light chains of the immunoprecipitating antibody using conventional HRP-conjugated anti-mouse antibody are detected but not using Mouse TrueBlot® ULTRA: Anti-Mouse Ig HRP. When the group using Mouse TrueBlot® ULTRA is re-immunoblotted using conventional HRP-conjugated anti-mouse polyclonal antibody, the heavy and light chains are now detected, confirming that although the immunoprecipitating heavy and light chains are present, Mouse TrueBlot® ULTRA: Anti-Mouse Ig HRP detects only native antibody and not denatured heavy and light chains.
Rabbit TrueBlot®: Anti-Rabbit IgG HRP: Rockland Immunochemicals, Cat No. 18-8816-31. Rabbit TrueBlot® IP / Western Blot: Jurkat cell lysate (0.5 ml of 1x10e7 cells/ml) was incubated with rabbit anti-human Stat1 and immunoprecipitated using Protein G, Protein A and Anti-Rabbit Ig IP Beads, subjected to electrophoresis, transferred to a PVDF membrane, and western blotted with anti-Stat1 using Rabbit TrueBlot®: Anti-Rabbit IgG HRP. Compared with groups using Protein G and Protein A beads, using Rabbit TrueBlot®: Anti-Rabbit IgG HRP with Anti-Rabbit Ig IP Beads shows a single band for Stat1, indicating that Rabbit TrueBlot®: Anti-Rabbit IgG HRP detects only native antibody and not denatured heavy and light chains.

Eukaryotic cell lines

Policy information about cell lines

Cell line source(s)	A549, MCF-7, HCT116, MDA-MB-231, U2OS and HME-1 cell lines were purchased from the Global Bioresource Center (ATCC). MCF10A cell line was originally generated by the Debnath lab (Harvard Medical School).
Authentication	Cell line authenticity was confirmed using the AmpFISTR Identifiler PCR Amplification Kit from Applied Biosystems and GeneMarker V1.91 software (SoftGenetics LLC).
Mycoplasma contamination	All cells used in this study were tested negative for mycoplasma contamination.
Commonly misidentified lines (See ICLAC register)	No commonly misidentified cell lines were used.

Animals and other organisms

Policy information about studies involving animals; ARRIVE guidelines recommended for reporting animal research

Laboratory animals	nu/nu nude mice, female, 4-week old were purchased from Shanghai SLAC Laboratory Animal Co.,Ltd. All the mice were housed in a temperature-controlled room (21-23 °C) with 40–60% humidity and a light/dark cycle of 12 h/12 h.
Wild animals	This study did not involve wild animals.
Field-collected samples	This study did not involve samples collected from the field.
Ethics oversight	All animal studies were conducted in accordance with relevant guidelines and regulations and were approved by the Animal Research Ethics Committee of the Shanxi Cancer Hospital.

Note that full information on the approval of the study protocol must also be provided in the manuscript.

Human research participants

Policy information about studies involving human research participants

Population characteristics	The tissue microarray of a cohort of 88 lung cancer with paired normal tissues were purchased from Shanghai Outdo Biotech Co., Ltd. 67 out of 88 patients have the information of clinicopathological characteristics. This cohort were composed of 39 males and 28 females with median age of 63 years old.<br>The tissue microarray of a cohort of 87 colon cancer with paired normal tissues were purchased from Shanghai Outdo Biotech Co., Ltd. 84 out of 87 patients have the information of clinicopathological characteristics. This cohort were composed of 48 males and 36 females with median age of 68 years old.<br>A cohort of 15 colon adnoma tissues and 16 normal colon tissues were collected at the Shanxi Cancer Hospital, and the covariate-relevant characteristics were not recorded as it is irrelavent to this study.
Recruitment	Patients are randomly recruited. No self-selection bias or other biases are present.
Ethics oversight	All human studies were conducted in accordance with relevant guidelines and regulations and were approved by the Human Research Ethics Committee of the Shanxi Cancer Hospital.

Note that full information on the approval of the study protocol must also be provided in the manuscript.

Flow Cytometry

Plots

Confirm that:

☒ The axis labels state the marker and fluorochrome used (e.g. CD4-FITC).

☒ The axis scales are clearly visible. Include numbers along axes only for bottom left plot of group (a 'group' is an analysis of identical markers).

☒ All plots are contour plots with outliers or pseudocolor plots.

☒ A numerical value for number of cells or percentage (with statistics) is provided.

Methodology

Sample preparation	Cell cycle analysis: Cells were fixed by 70% Ethanol on ice for 1 hour and spun down at 1500 × g. Cell pellets were re-suspended in PBS containing 0.25% Triton X-100 and incubate on ice for 15 min. Discard supernatant and re-suspend cell pellet in 0.5 ml PBS containing 10 μg/ml RNase A and 20 μg/ml PI stock solution and incubate at room temperature (RT) in the dark for 30 min. Cells were then subjected to analysis using a flow cytometer (FACSCanto, BD Biosciences).<br>Apoptosis analysis: Apoptotic cells were quantitated using the FITC Annexin V Apoptosis Detection Kit (BD Biosciences, Cat#556547) according to the manufacturer's instructions. In brief, cells resuspended in binding buffer were incubated with Annexin V/propidium iodide (PI) for 15 min at room temperature in dark before analysis using a flow cytometer (FACSCanto II, BD Biosciences).
Instrument	BD FACSCanto flow cytometer.
Software	BD FACSDiva V8.0.1 software
Cell population abundance	No sorting was employed.
Gating strategy	Cells were gated based on forward and side scatter plots, only avoiding debris and aggregates and no extensive gating strategy was used.

☒ Tick this box to confirm that a figure exemplifying the gating strategy is provided in the Supplementary Information.
